# Assessment of Combination Therapies vs Monotherapy for Erectile Dysfunction: A Systematic Review and Meta-analysis

**DOI:** 10.1001/jamanetworkopen.2020.36337

**Published:** 2021-02-18

**Authors:** Ioannis Mykoniatis, Nikolaos Pyrgidis, Ioannis Sokolakis, Andreas Ouranidis, Petros Sountoulides, Anna-Bettina Haidich, Koenraad van Renterghem, Georgios Hatzichristodoulou, Dimitrios Hatzichristou

**Affiliations:** 1 Department of Urology, Faculty of Medicine, Aristotle University of Thessaloniki School of Health Sciences, Thessaloniki, Greece; 2 Department of Urology, Martha-Maria Hospital Nuremberg, Nuremberg, Germany; 3 Department of Pharmaceutical Technology, Aristotle University of Thessaloniki, Thessaloniki, Greece; 4 Department of Chemical Engineering, Aristotle University of Thessaloniki, Thessaloniki, Greece; 5 Department of Hygiene, Social-Preventive Medicine & Medical Statistics, Aristotle University of Thessaloniki Medical School, University Campus, Thessaloniki, Greece; 6 Department of Urology, Jessa Hospital, Hasselt University, Hasselt, Belgium; 7 Department of Urology, University Hospitals Leuven, Leuven, Belgium

## Abstract

**Importance:**

Combining 2 first-line treatments for erectile dysfunction (ED) or initiating other modalities in addition to a first-line therapy may produce beneficial outcomes.

**Objective:**

To assess whether different ED combination therapies were associated with improved outcomes compared with first-line ED monotherapy in various subgroups of patients with ED.

**Data Sources:**

Studies were identified through a systematic search in MEDLINE, Cochrane Library, and Scopus from inception of these databases to October 10, 2020.

**Study Selection:**

Randomized clinical trials or prospective interventional studies of the outcomes of combination therapy vs recommended monotherapy in men with ED were identified. Only comparative human studies, which evaluated the change from baseline of self-reported erectile function using validated questionnaires, that were published in any language were included.

**Data Extraction and Synthesis:**

Data extraction and synthesis were performed according to the Preferred Reporting Items for Systematic Reviews and Meta-analyses (PRISMA) reporting guideline.

**Main Outcomes and Measures:**

A meta-analysis was conducted that included randomized clinical trials that compared outcomes of combination therapy with phosphodiesterase type 5 (PDE5) inhibitors plus another agent vs PDE5 inhibitor monotherapy. Separate analyses were performed for the mean International Index of Erectile Function (IIEF) score change from baseline and the number of adverse events (AEs) by different treatment modalities and subgroups of patients.

**Results:**

A total of 44 studies included 3853 men with a mean (SD) age of 55.8 (11.9) years. Combination therapy compared with monotherapy was associated with a mean IIEF score improvement of 1.76 points (95% CI, 1.27-2.24; *I*^2^ = 77%; 95% PI, −0.56 to 4.08). Adding daily tadalafil, low-intensity shockwave therapy, vacuum erectile device, folic acid, metformin hydrochloride, or angiotensin-converting enzyme inhibitors was associated with a significant IIEF score improvement, but each measure was based on only 1 study. Specifically, the weighted mean difference (WMD) in IIEF score was 1.70 (95% CI, 0.79-2.61) for the addition of daily tadalafil, 3.50 (95% CI, 0.22-6.78) for the addition of low-intensity shockwave therapy, 8.40 (95% CI, 4.90-11.90) for the addition of a vacuum erectile device, 3.46 (95% CI, 2.16-4.76) for the addition of folic acid, 4.90 (95% CI, 2.82-6.98) for the addition of metformin hydrochloride and 2.07 (95% CI, 1.37-2.77) for the addition of angiotensin-converting enzyme inhibitors. The addition of α-blockers to PDE5 inhibitors was not associated with improvement in IIEF score (WMD, 0.80; 95% CI, −0.06 to 1.65; *I*^2^ = 72%). Compared with monotherapy, combination therapy was associated with improved IIEF score in patients with hypogonadism (WMD, 1.61; 95% CI, 0.99-2.23; *I*^2^ = 0%), monotherapy-resistant ED (WMD, 4.38; 95% CI, 2.37-6.40; *I*^2^ = 52%), or prostatectomy-induced ED (WMD, 5.47; 95% CI, 3.11-7.83; *I*^2^ = 53%). The treatment-related AEs did not differ between combination therapy and monotherapy (odds ratio, 1.10; 95% CI, 0.66-1.85; *I*^2^ = 78%). Despite multiple subgroup and sensitivity analyses, the levels of heterogeneity remained high.

**Conclusions and Relevance:**

This study found that combination therapy of PDE5 inhibitors and antioxidants was associated with improved ED without increasing the AEs. Treatment with PDE5 inhibitors and daily tadalafil, shockwaves, or a vacuum device was associated with additional improvement, but this result was based on limited data. These findings suggest that combination therapy is safe, associated with improved outcomes, and should be considered as a first-line therapy for refractory, complex, or difficult-to-treat cases of ED.

## Key Points

**Question:**

Are different combination therapies associated with improved outcomes compared with first-line monotherapy in various subgroups of individuals with erectile dysfunction?

**Findings:**

This systematic review and meta-analysis of 44 studies with 3853 men found that combination therapy with phosphodiesterase type 5 inhibitors and antioxidants was associated with improved erectile dysfunction without increasing the number of adverse events. Treatment with phosphodiesterase type 5 inhibitors and daily tadalafil, low-intensity shockwave therapy, or a vacuum erectile device were associated with additional improvement based on limited data.

**Meaning:**

Results of this study suggest that combination therapy should be considered as a first-line treatment for difficult-to-treat cases of erectile dysfunction.

## Teaser

This systematic review and meta-analysis compares the outcomes of monotherapy and combination treatments according to self-reports of erectile function by men with erectile dysfunction.

## Introduction

Erectile dysfunction (ED) has an increasing worldwide prevalence and is associated with a complex, pathophysiological mechanism.^1,2^ In patients with primary organic causes, phosphodiesterase type 5 (PDE5) inhibitors are considered the first-line monotherapy because of their safety profile, rapid efficacy, and convenient oral administration.^3^ Other recommended first-line treatment modalities include intracavernosal injections, topical or intraurethral alprostadil, vacuum erectile device, and low-intensity extracorporeal shockwave therapy (Li-ESWT).^4^

PDE5 inhibitors and other first-line ED treatments provide great initial benefits for most patients.^4,5,6^ Switching among first-line ED treatments may prove helpful for some nonresponders.^4,5,6^ However, more than half of the patients have reported dissatisfaction, presented low adherence rates, or even abandoned the first-line ED therapeutic options because of lack of efficacy, inconvenient administration, adverse events (AEs), or contraindications.^7,8,9^ Before considering penile prosthesis implant, individuals may use a combination of 2 or more first-line ED treatments or other modalities in addition to first-line ED treatments, which seems, in this context, to be associated with beneficial outcomes.^10^ Moreover, in some individuals with curable causes of ED, such as hypogonadism, the coadministration of ED treatments and population-targeted therapies, such as testosterone, may prove successful.^11^ On the other hand, the benefit of combination therapy may inevitably come at the cost of more treatment-related AEs.^12,13^

Within this framework, we generated a systematic review and meta-analysis to compare the outcomes of different ED combination therapies with those of first-line monotherapy in various subgroups of patients with ED.

## Methods

### Data Sources and Searches

We predefined the objectives and methods in a protocol registered at PROSPERO (CRD42020193401), revised some methods based on editor and peer review comments, and followed the Preferred Reporting Items for Systematic Reviews and Meta-analyses (PRISMA) reporting guideline.^14^ Two of us (I.M. and N.P.) conducted a systematic search in MEDLINE, Scopus, and Cochrane Library from inception of these databases to October 10, 2020. In addition, we hand-searched sources of gray literature, including clinical trial registries and conference abstracts published in major urological and sexual medicine journals. We perused the reference lists of all eligible studies as well as relevant reviews. The detailed search syntax and search string are presented in eAppendix 1 in the Supplement.

### Selection Criteria

We included randomized clinical trials (RCTs) or prospective interventional studies of the outcomes of combination therapy vs recommended monotherapy (PDE5 inhibitors, intracavernosal injections, topical or intraurethral alprostadil, vacuum erectile device, or Li-ESWT) among men with ED. We considered only comparative human studies, which evaluated the change from baseline of self-reported erectile function using validated questionnaires, that were published in any language. Conversely, studies that compared combination therapy with placebo or with a nonrecommended monotherapy were excluded. Accordingly, we did not include articles that evaluated the treatment of psychogenic ED with psychosocial and behavioral interventions. Similarly, we did not consider studies of the role of acupuncture or traditional Chinese medicine. Studies that evaluated combination treatment in patients with Peyronie disease and phase 1 clinical trials were also excluded. When multiple records with potential overlapping populations were identified, only the most recent study was included.

### Data Extraction and Risk-of-Bias Assessment

Two of us (I.M. and N.P.) assessed the titles and abstracts of all retrieved articles. Full text of potentially eligible articles was evaluated according to the selection criteria. Data extraction was performed independently in a predesigned spreadsheet (Microsoft Excel; Microsoft Corp). For each included article, we tabulated study and participant characteristics data as well as outcomes of combination therapy and monotherapy. Any disagreements were resolved by consensus between 2 of us (I.M. and N.P.).

In studies that assessed erectile function at multiple time points, only baseline and last evaluation data were extracted. Similarly, in studies of the outcome of different therapeutic doses, we used only the data from patients assigned to the highest available approved dose. When the SD for the mean erectile function change from baseline was not reported, the SD was obtained from the relevant SE, CI, or *P* value.^15^ When not enough data were available to calculate SDs, the data were imputed from the correlation coefficient reported in other included trials.^15^ With a sensitivity analysis of different values of correlation coefficient, we found that the overall result of the analysis did not change by the imputed SDs. Regarding AEs, we initiated an exploratory approach by synthesizing the data on any AEs reported in the included studies. Study authors were directly contacted for missing data or further information.

To evaluate the risk of bias in each study, we used the RoB-2, a risk-of-bias tool, for RCTs^16^ and the Robins–I tool for nonrandomized trials.^17^ Discrepancies were resolved through consensus between 2 of us (I.M. and N.P.). Accordingly, we assessed the publication bias and small-study bias by a visual assessment of funnel plot asymmetry and by the Egger test.^18^

### Data Synthesis, Statistical Analysis, and Grading of Evidence

Because of the scarcity of identified non–PDE5 inhibitor monotherapies and combination therapies, we performed an inverse variance random effects meta-analysis that included RCTs that compared the combination treatment of PDE5 inhibitors plus another agent with the PDE5 inhibitor monotherapy. We calculated the weighted mean differences (WMDs) for the mean International Index of Erectile Function (IIEF) score change and the odds ratios for the number of AEs with the corresponding 95% CIs and 95% prediction intervals (PIs). The PIs aim to estimate the interval of the observed treatment outcome of future relevant studies.^15^ We performed separate analyses for the mean IIEF score change and the number of AEs by different treatment modalities and subgroups of patients with ED. For the mean IIEF score change, we undertook a subgroup analysis with RCTs that included responders or nonresponders to PDE5 inhibitors. Accordingly, we conducted sensitivity analyses with placebo-controlled RCTs and with studies at low risk of bias.

Heterogeneity was estimated with the *I*^2^, and its statistical significance was calculated with the Cochran *Q* test.^19^ All statistical analyses were performed with the meta package in R, version 3.6.3 (R Foundation for Statistical Computing).

We used the GRADE approach to ascertain the overall strength of evidence across the trials.^20^ Two of us (I.M. and N.P.) graded risk of bias, inconsistency, indirectness, imprecision, and publication bias for the mean IIEF score change from baseline and number of AEs.

## Results

A total of 44 studies with 3853 men were included in the qualitative synthesis.^21,22,23,24,25,26,27,28,29,30,31,32,33,34,35,36,37,38,39,40,41,42,43,44,45,46,47,48,49,50,51,52,53,54,55,56,57,58,59,60,61,62,63,64^ The mean (SD) patient age was 55.8 (11.9) years. Participants were followed up for a mean (SD) duration of 13.6 (7.8) weeks and had a mean ED duration of 2.9 years. All study authors assessed ED at baseline and at the end of each study follow-up using the erectile function domain of the IIEF-15 (IIEF-ED)^65^ or the IIEF-5 questionnaire.^66^ Except for 1 trial that assessed Li-ESWT vs platelet-rich plasma,^62^ all other studies evaluated the outcomes of PDE5 inhibitors as part of combination therapy and/or monotherapy.^21,22,23,24,25,26,27,28,29,30,31,32,33,34,35,36,37,38,39,40,41,42,43,44,45,46,47,48,49,50,51,52,53,54,55,56,57,58,59,60,61,63,64^ Across the included trials, sildenafil citrate and tadalafil were the preferred PDE5 inhibitors.^21,22,23,24,25,26,27,28,29,30,31,32,33,34,35,36,37,38,39,40,41,42,43,44,45,46,47,48,49,50,51,52,53,54,55,56,57,58,59,60,61,63,64^ Some studies examined PDE5 inhibitors as a continuous daily, low-dose drug,^21,23,24,25,27,28,29,30,33,39,40,41,42,56,58,63^ whereas other studies evaluated it as an on-demand, high-dose agent taken prior to intercourse.^22,26,31,32,34,35,36,37,38,43,44,45,46,47,48,49,50,51,52,53,54,55,57,59,60,61,64^ The study selection process is illustrated in eAppendix 2 and 3 in the Supplement, and the characteristics of all included studies are described in the Table.

**Table.** Characteristics of All Included Studies^a^

Source; study location	Study design	Population	Combination therapy	Monotherapy	Participants, No.	Follow-up, wk	Mean (SD), y		Adverse events
							Participant age	ED duration	
Abolyosr et al,^21^ 2013; Egypt	Open-label RCT	Patients with BPH-related LUTS + ED	Sildenafil citrate 50 mg/d + doxazosin mesylate 2 mg/d	Sildenafil 50 mg/d	100	16	NA	NA	Slight dizziness and blurring, especially in active group. No AE-related dropouts were reported in both groups.
Aversa et al,^22^ 2003; Italy	Placebo-controlled RCT	Nonresponders to PDE5i	Sildenafil 100 mg on demand + transdermal testosterone patch 5 mg/d	Sildenafil 100 mg on demand + transdermal placebo patch	20	4	Active: 54.0 (2.0) Control: 56.0 (4.0)	NA	No noticeable AEs or AE-related dropouts were reported in both groups.
Baccaglini et al,^23^ 2020; Brazil	Open-label RCT	Patients with induced ED after open or laparoscopic radical prostatectomy	Tadalafil 5 mg/d +8 sessions of Li-ESWT (once/wk)	Tadalafil 5 mg/d	92	16	Active: 64.6 (5.3) Control: 64.6 (5.3)	NA	No AEs were reported during Li-ESWT. No AE-related dropouts were reported in both groups.
Bayraktar and Albayrak,^24^ 2019; Turkey	Open-label RCT	Patients with ED	Tadalafil 5 mg/d + aspirin 100 mg/d	Tadalafil 5 mg/d	144	6	Active: 47.1 (14.3) Control: 46.5 (13.7)	NA	Active: dyspepsia: 5; headache: 3; flushing: 2. Control: headache: 10; flushing: 7; dyspepsia: 4. 2 AE-related dropouts in the active group and 5 in the control group.
Buvat et al,^25^ 2011; France, Italy, Germany, Finland, Spain, Holland, UK, and US	Placebo-controlled RCT	Patients with hypogonadism + nonresponders to PDE5i	Tadalafil 10 mg/d + testosterone gel 50-100 mg/d	Tadalafil 10 mg/d + placebo gel	173	12	Active: 58.3 (7.56) Control: 59.9 (7.38)	Active: 5.1 (4.4) Control: 5.9 (4.9)	No serious AEs related to treatment in both groups. 4 AE-related dropouts in the active group and 7 in the control group.
Cavallini et al,^26^ 2005; Italy	Placebo-controlled RCT	Patients with induced ED after radical prostatectomy	Sildenafil 100 mg on demand + propionyl-l-carnitine 2 g/d + acetyl-l-carnitine 2 g/d	Sildenafil 100 mg on demand + placebo	77	16	Active: 63.0 (3.9) Control: 61.0 (4.4)	Active: 1.3 (0.33) Control: 1.2 (0.36)	Active: headache: 8; flushing: 7; dizziness: 3. Control: headache: 9; flushing: 8; dizziness: 3. No AE-related dropouts were reported in both groups.
Chen et al,^53^ 2004; Israel	Pre-/postprospective open-label RCT	Nonresponders to monotherapy who subsequently received combination therapy	Sildenafil 100 mg/d + VED	Sildenafil 100 mg or VED in a crossover design for nonresponders	161	24	NA	NA	No AE-related dropouts were reported in both groups.
Chen et al,^27^ 2012; China	Open-label RCT	Patients with diabetic ED	Tadalafil 5 mg/d + losartan potassium 50 mg/d	Tadalafil 5 mg/d	62	12	Active: 45.4 (14.9) Control: 46.1 (12.9)	Active: 2.1 (0.97) Control: 2.2 (0.98)	Active: hypotension: 2; dizziness: 2; headache: 1. Control: headache: 1; rhinorrhagia: 1. No AE-related dropouts were reported in both groups.
Cui et al,^28^ 2015; China	Open-label RCT	Patients with ED	Tadalafil 5 mg/d + sildenafil 50 mg on demand	Tadalafil 5 mg/d	180	12	Active: 32.4 (10.6) Control: 33.4 (9.9)	Active: 1.2 (1.59) Control: 1.1 (0.98)	Active: flushing: 7; headache: 4; dyspepsia: 4. Control: flushing: 6; headache: 5; muscle pain: 4. No AE-related dropouts were reported in both groups.
El Taieb et al,^29^ 2019; Egypt	Placebo-controlled RCT	Patients with diabetic ED	Tadalafil 10 mg/d + l-arginine 5 g/d	Tadalafil 10 mg/d + placebo	54	8	Active: 45.3 (4.3) Control: 43.0 (5.9)	Active: 4.4 (0.3) Control: 5.0 (0.5)	No AE-related dropouts were reported in both groups.
Abu El-Hamd and Hegazy,^30^ 2020; Egypt	Placebo-controlled RCT	Patients with ED aged ≥60 y	Tadalafil 5 mg/d + l-arginine 5 g/d	Tadalafil 5 mg/d + placebo	60	6	Active: 66.3 (6.13) Control: 66.1 (6.1)	Active: 4.3 (1.95) Control: 4.2 (1.92)	No AE-related dropouts were reported in both groups.
El-Wakeel et al,^31^ 2020; Egypt	Open-label RCT	Sexually active male patients older than 50 y with organic ED	Sildenafil 50 mg on demand + l-arginine 3 g/d	Sildenafil 50 mg on demand	70	8	Active: 56.2 (4.4) Control: 56.3 (5.1)	Active: 1.1 (1.89) Control: 1.9 (2.16)	Mild, similar AEs on both groups, except for gastritis, which was more common in the active group. No AE-related dropouts were reported in both groups.
Engel,^32^ 2011; US	Open-label RCT	Patients with induced ED after bilateral nerve-sparing robotic radical prostatectomy	Tadalafil 20 mg 3 times/wk + VED at least 5 times/wk	Tadalafil 20 mg 3 times/wk	23	48	NA	NA	AEs after tadalafil were headache, flushing, and muscle ache, and AEs after VED were minor local discomfort. 3 Dropouts in the control group because of lack of efficacy or adverse effects were reported.
Gallo et al,^33^ 2020; Italy	Open-label RCT	Patients with ED	Tadalafil 5 mg/d + l-arginine 2.5 g/d	Tadalafil 5 mg/d	200	12	Active: 56.7 (9.9) Control: 56.2 (9.8)	NA	Active: dyspepsia: 14; headache: 11; myalgia: 9. Control: dyspepsia: 11; headache: 8; myalgia: 8. No AE-related dropouts were reported in both groups.
Gentile et al,^34^ 2004; Italy	Placebo-controlled RCT	Patients with diabetic ED	Sildenafil 50 mg twice/wk + propionyl-l-carnitine 2 g/d	Sildenafil 50 mg twice/wk + placebo	40	24	Active: 63.7 (4.5) Control: 64.1 (9.0)	Active: 5.7 (5.33) Control: 5.3 (6.3)	Active: mild gastric pain: 2. Control: none reported. No AE-related dropouts were reported in both groups.
Gutierrez et al,^54^ 2005; Spain	Placebo-controlled, crossover, prospective interventional study	Nonresponders to PDE5i	Sildenafil 50 mg on demand + IC PGE1 injections 20 μg/2 wk	IC PGE1 injections 20 μg/2 wk + placebo	40	8	NA	NA	No noticeable AEs or AE-related dropouts were reported in both groups.
Hamidi Madani et al,^35^ 2013; Iran	Placebo-controlled RCT	Patients with diabetic ED	Tadalafil 10 mg on demand + folic acid 5 mg/d	Tadalafil 10 mg on demand + placebo	83	12	Active: 55.7 (6.22) Control: 57.7 (5.98)	Active: 2.0 (1.5) Control: 1.6 (0.9)	Active: folic acid + tadalafil were well tolerated. Control: headache: 3; low back pain: 3; flushing: 1. No AE-related dropouts were reported in both groups.
Herrmann et al,^36^ 2006; US	Placebo-controlled RCT	Nonresponders to PDE5i	Sildenafil 100 mg on demand + atorvastatin 80 mg/d	Sildenafil 100 mg on demand + placebo	16	12	Active: 57.0 (14.0) Control: 61.0 (9.0)	Active: 4.2 (4.8) Control: 2.6 (2.9)	1 Dropout because of atorvastatin-related AE.
Hwang et al,^55^ 2006; Taiwan	Pre-/postprospective interventional study	Patients with hypogonadism + nonresponders to PDE5i	Sildenafil 100 mg on demand + oral testosterone undecanoate 160 or 240 mg/d	Sildenafil 100 mg on demand	32	16	NA	NA	No AE-related dropouts were reported in both groups.
Jin et al,^37^ 2011; China	Open-label RCT	Patients with BPH-related LUTS + ED	Sildenafil 25-100 mg on demand + doxazosin 4 mg/d	Sildenafil 25-100 mg on demand	250	24	Active: 61.7 (5.3) Control: 60.8 (5.9)	NA	Active: headache: 9; dizziness: 8; flushing: 6 Control: headache: 5; dizziness: 4; flushing: 4. No AE-related dropouts were reported in both groups.
Jung and Heo,^38^ 2008; South Korea	Open-label RCT	Patients with previously untreated LUTS + ED	Tadalafil 20 mg 3 times/wk + alfuzosin hydrochloride 10 mg/d	Tadalafil 20 mg 3 times/wk	101	12	NA	NA	No AE-related dropouts were reported in both groups.
Kaplan et al,^39^ 2007; US	Open-label RCT	Patients with moderate to severe untreated LUTS	Sildenafil 25 mg/d + alfuzosin 10 mg/d	Sildenafil 25 mg/d	42	12	Active: 63.1 (6.9) Control: 64.5 (5.9)	Active: 2.2 (0.45) Control: 2.1 (0.45)	No serious AEs reported during the study. 3 AE-related dropouts in the active group (gastric upset: 2; dizziness: 1) and 2 in the control group (flushing: 1; dyspepsia: 1).
Karami et al,^40^ 2016; Iran	Open-label RCT	Patients with BPH-related LUTS + ED	Tadalafil 20 mg/d + tamsulosin hydrochloride 0.4 mg/d	Tadalafil 20 mg/d	122	12	Active: 67.9 (8.8) Control: 68.2 (7.8)	NA	Active: myalgia: 4; back pain: 3; headache: 3. Control: back pain: 4; myalgia: 3; headache: 3. 3 AE-related dropouts in the active group and 1 in the control group.
Kim et al,^56^ 2013; South Korea	Pre-/postprospective interventional study	Patients with hypogonadism + ED	Tadalafil 5 mg/d + intramuscular testosterone enanthate/4 wk	Tadalafil 5 mg/d	46	24	NA	NA	No noticeable AEs or AE-related dropouts were reported in both groups.
Kim et al,^41^ 2017; South Korea	Placebo-controlled RCT	Patients with BPH-related LUTS + ED	Tadalafil 5 mg/d + tamsulosin 0.4 mg/d	Tadalafil 5 mg/d + placebo	315	12	Active: 61.8 (5.71) Control: 61.9 (6.83)	NA	Active: headache: 8; nasal congestion: 5; ocular hyperemia: 5. 2 Serious AEs. Control: headache: 3; gastritis: 2; flushing: 1. 1 Serious AE. No AE-related dropouts were reported in both groups.
Kumar et al,^42^ 2015; India	Open-label RCT	Patients with ED	Tadalafil 10 mg/d + pentoxifylline 1200 mg/d	Tadalafil 10 mg/d	237	8	Active: 47.0 (7.0) Control: 46.7 (6.7)	NA	Active: headache: 11; back pain: 3; nasal stuffiness: 2. Control: headache: 9; back pain: 4; nasal stuffiness: 2. No AE-related dropouts were reported in both groups.
Law et al,^43^ 2020; Singapore	Placebo-controlled RCT	Patients with ED	Sildenafil 100 mg on demand + pentoxifylline 1200 mg/d	Sildenafil 100 mg on demand + placebo	58	8	Active: 59.3 (12.85) Control: 60.1 (8.03)	NA	Active: gastrointestinal: 4; neurological: 4; musculoskeletal: 2. Control: gastrointestinal: 2; neurological: 1. 3 AE-related dropouts in the active group and 1 in the control group.
Liguori et al,^44^ 2009; Italy	Open-label RCT	Patients with BPH-related LUTS + ED	Tadalafil 20 mg on demand + alfuzosin 10 mg/d	Tadalafil 20 mg on demand	44	12	Active: 61.5 (5.8) Control: 60.8 (8.0)	NA	No severe AEs were reported during the study. 2 AE-related dropouts in the active group (myalgia, dizziness, and sensation of heaviness) and 1 in the control group (back pain and headaches).
McMahon et al,^57^ 1999; Australia	Pre-/postprospective interventional study	Nonresponders to IC injections	Sildenafil 100 mg on demand + IC injections of alprostadil, papaverine, and phentolamine mesylate on demand	Sildenafil 100 mg on demand	93	NA	NA	NA	Active: headache: 15; penile pain: 15; flushing 12. Control: headache: 30; flushing: 25; dyspepsia: 12. 4 AE-related dropouts in the active group (headache, dizziness, dyspepsia, and flushing) and 3 in the control group (headache and dyspepsia).
Morano et al,^45^ 2007; Italy	Placebo-controlled RCT	Patients with diabetic ED	Sildenafil 50 mg twice/wk + propionyl-l-carnitine 2 g/d	Sildenafil 50 mg twice/wk + placebo	16	12	Active: 57.8 (7.0) Control: 54.0 (7.4)	NA	No AE-related dropouts were reported in both groups.
Nandipati et al,^58^ 2006; US	Pre-/postprospective interventional study	Patients with induced ED after bilateral nerve-sparing robotic radical prostatectomy	Sildenafil 50 mg/d + IC injections of alprostadil or mix of alprostadil, papaverine, and phentolamine on demand	IC injections of alprostadil or mix of alprostadil, papaverine, and phentolamine on demand	22	24	NA	NA	IC injections: penile discomfort: 2. Sildenafil: headache: 2. No AE-related dropouts were reported in both groups.
Ozdal et al,^59^ 2008; Turkey	Pre-/postprospective interventional study	Patients with ED	Sildenafil on demand + pentoxifylline 1200 mg/d	Sildenafil on demand	68	8	NA	NA	No noticeable AEs or AE-related dropouts were reported in both groups.
Palmieri et al,^60^ 2020; Italy	Pre-/postprospective interventional study	Nonresponders to PDE5i + at least 1 cardiovascular risk factor	6 Sessions of Li-ESWT (twice/wk) + maximum dose of on demand or daily PDE5i	Maximum dose on demand or daily PDE5i	109	4	57.9 (10.7)	2.8 (2.4)	1 Patient developed Peyronie disease.
Raina et al,^61^ 2005; US	Pre-/postprospective open-label RCT	Nonresponders to monotherapy who subsequently received combination therapy	Sildenafil 100 mg on demand + VED on demand	VED on demand	72	NA	NA	NA	No AE-related dropouts were reported in both groups.
Rey-Valzacchi et al,^46^ 2012; Argentina	Placebo-controlled RCT	Nonresponders to PDE5i + nondiabetic, insulin-resistant patients	Sildenafil 100 mg on demand + metformin hydrochloride 1700 mg/d	Sildenafil 100 mg on demand + placebo	30	16	Active: 65.7 (5.2) Control: 62.6 (6.6)	NA	Active: mild gastrointestinal AEs: 9. Control: mild gastrointestinal AEs: 1. No AE-related dropouts were reported in both groups.
Ruffo et al,^62^ 2020; Italy	Open-label RCT	Patients with ED	6 sessions of Li-ESWT (once/wk) + 3 IC injections of PRP (once/2 wk)	6 sessions of Li-ESWT (once/wk)	112	12	NA	NA	No AEs or AE-related dropouts were reported in both groups.
Sebastianelli et al,^63^ 2019; Italy	Placebo-controlled, prospective interventional study	Patients with BPH-related LUTS + ED	Tadalafil 5 mg/d + tamsulosin hydrochloride 0.4 mg/daily	Tadalafil 5 mg/d + placebo	75	12	Active: 65.7 (9.1) Control: 65.5 (6.3)	NA	Active: headache: 4; back pain: 3; ejaculatory dysfunction: 2. Control: headache: 2; nasopharyngitis: 1; back pain: 1. No serious AEs or AE-related dropouts were reported in both groups.
Shabsigh et al,^47^ 2004: US	Placebo-controlled RCT	Patients with hypogonadism + nonresponders to PDE5i	Sildenafil 100 mg on demand + transdermal testosterone gel 5 g/d	Sildenafil 100 mg on demand + transdermal placebo gel	75	12	Active: 56.8 (10.2) Control: 59.1 (9.4)	NA	1 AE-related dropout in the active group and none in the control group.
Shamloul et al,^48^ 2005; Egypt	Open-label RCT	Patients with hypogonadism + partial responders to PDE5i	Sildenafil on demand + oral testosterone undecanoate 120 mg/d	Sildenafil on demand	20	8	NA	NA	3 Patients on sildenafil reported mild headache. No AE-related dropouts were reported in both groups.
Shirai et al,^49^ 2018; Japan	Placebo-controlled, crossover RCT	Nonresponders to PDE5i	PDE5i on demand + l-citrulline 800 mg/d + transresveratol 300 mg/d	PDE5i on demand + placebo	20	8	NA	NA	13 Patients completed the study without AEs.
Spitzer et al,^50^ 2012; US	Placebo-controlled RCT	Patients with hypogonadism + ED	Sildenafil on demand + transdermal testosterone gel up to 15 g/d	Sildenafil on demand + transdermal placebo gel	140	14	Active: 55.1 (8.3) Control: 54.6 (8.5)	NA	Active: respiratory: 14; musculoskeletal: 14; dermatologic: 12. 2 Serious AEs. Control: dermatologic: 15; respiratory: 14; musculoskeletal: 14. 4 Serious AEs. 5 AE-related dropouts in the active group and 1 in the control group.
Sun et al,^64^ 2014; China	Open-label RCT	Diabetic nonresponders to PDE5i	Sildenafil 100 mg/d + VED on demand	VED on demand	66	12	Active: 43.0 (9.5) Control: 45.0 (9.2)	Active: 2.63 (1.89) Control: 2.85 (1.77)	All AEs were mostly mild. Sildenafil: flushing: 6; nausea: 5; headache: 5. VED: penile bruising: 6; numbness: 5. No AE-related dropouts were reported in both groups.
Tuncel et al,^51^ 2010; Turkey	Open-label RCT	Patients with BPH-related LUTS + ED	Sildenafil 25 mg 4 d/wk + tamsulosin 0.4 mg/d	Sildenafil 25 mg 4 d/wk	40	8	NA	NA	No AE-related dropouts were reported in both groups.
Vicari et al,^52^ 2010; Italy	Open-label, crossover RCT	Patients with diabetic ED	Sildenafil 100 mg twice/wk + l-arginine, propionyl-l-carnitine, and nicotinic acid once/d	Sildenafil 100 mg twice/wk	53	24	NA	NA	Active: headache: 4; dyspepsia: 1. Control: headache: 14; flushing: 12; dyspepsia: 3. The frequency of sildenafil AEs was reduced during combination therapy.

Abbreviations: AE, adverse event; BPH, benign prostatic hyperplasia; ED, erectile dysfunction; IC, intracavernosal; Li-ESWT, low-intensity extracorporeal shock wave therapy; LUTS, lower urinary tract symptoms; NA, not available; PDE5i, phosphodiesterase type 5 inhibitors; PGE1, prostaglandin E1; PRP, platelet-rich plasma; RCT, randomized clinical trial; VED, vacuum erectile device.

^a^ Data are presented as mean (SD).

### Risk of Bias and Publication Bias

The overall risk of bias was low in 12 RCTs,^23,25,26,29,33,34,41,43,45,47,49,50^ with some concerns found in 16 RCTs^21,22,27,28,30,35,38,39,44,46,48,51,52,53,62,64^ and high risk of bias in 8 RCTs^24,31,32,36,37,40,42,61^ (eAppendix 4 in the Supplement). Accordingly, 3 non-RCTs^54,57,63^ were considered as having low risk of bias and 5 non-RCTs^54,56,58,59,60^ as having moderate risk of bias (eAppendix 5 in the Supplement). Funnel plot inspection and Egger test indicated potential publication bias and small-study bias (eAppendix 6 in the Supplement).

### Treatment Modalities

In the meta-analysis, we included 32 RCTs that compared the combination treatment of PDE5 inhibitors plus another agent with PDE5 inhibitors monotherapy. A total of 1428 participants were treated with a combination of PDE5 inhibitors plus another agent, and 1360 participants received PDE5 inhibitors monotherapy.^21,22,23,24,25,26,27,28,29,30,31,32,33,34,35,36,37,38,39,40,41,42,43,44,45,46,47,48,49,50,51,52^ Combination therapy compared with monotherapy was associated with a mean IIEF score improvement of 1.76 points (95% CI, 1.27-2.24; *I*^2^ = 77%; 95% PI, −0.56 to 4.08). The addition of testosterone to PDE5 inhibitors was associated with a mean IIEF score improvement of 2.27 points (95% CI, 0.9-3.65; *I*^2^ = 71), and the addition of antioxidants was associated with an improvement of 1.99 points (95% CI, 1.34-2.63; *I*^2^ = 59%). Combining PDE5 inhibitor treatment with daily tadalafil, Li-ESWT, vacuum erectile device, folic acid, metformin hydrochloride, or angiotensin-converting enzyme inhibitors was associated with significantly increased mean IIEF score compared with PDE5 inhibitors monotherapy, but each measure was based on only 1 study. Specifically, the weighted mean difference (WMD) in IIEF score for the addition of daily tadalafil was 1.70 (95% CI, 0.79-2.61), 3.50 (95% CI, 0.22-6.78) for the addition of low-intensity shockwave therapy, 8.40 (95% CI, 4.90-11.90) for the addition of a vacuum erectile device, 3.46 (95% CI, 2.16-4.76) for the addition of folic acid, 4.90 (95% CI, 2.82-6.98) for the addition of metformin hydrochloride and 2.07 (95% CI, 1.37-2.77) for the addition of angiotensin-converting enzyme inhibitors.

**Figure 1.**
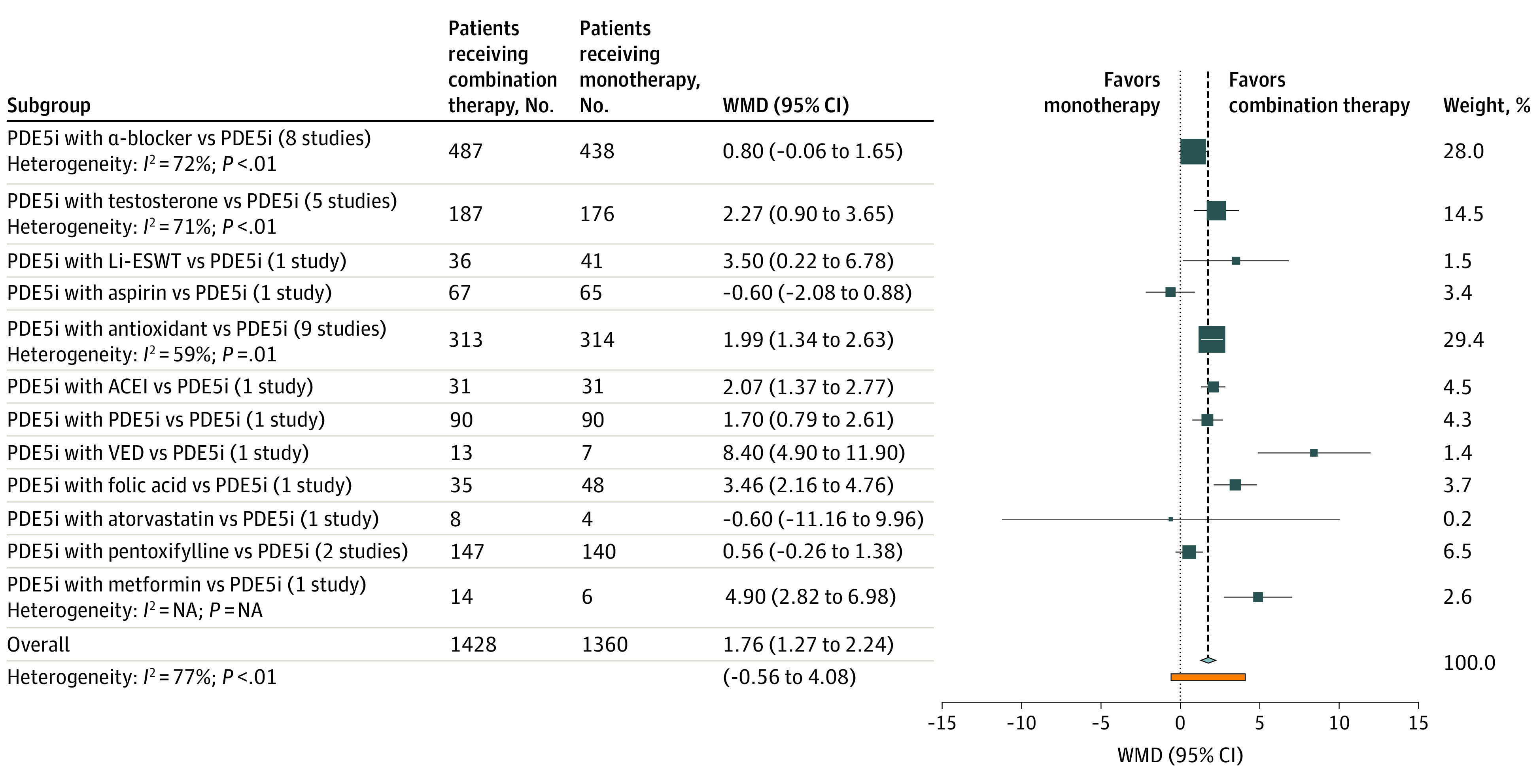
Forest Plot of the Mean Difference in International Index of Erectile Function (IIEF) Score of Different Combination Therapies vs Phosphodiesterase Type 5 inhibitors (PDE5i) Monotherapy ACEI indicates angiotensin-converting enzyme inhibitor; Li-ESWT, low-intensity extracorporeal shockwave therapy; VED, vacuum erectile device; and WMD, weighted mean difference.

In contrast, the mean IIEF score did not improve significantly with the addition of α-blockers (WMD, 0.80; 95% CI, −0.06 to 1.65; *I*^2^ = 72%) or pentoxifylline (WMD, 0.56; 95% CI, −0.26 to 1.38; *I*^2^ = 4%) to PDE5 inhibitors. The comparison of the outcomes of all combination modalities vs PDE5 inhibitor monotherapy is presented in Figure 1 and eAppendix 7 in the Supplement. Among the studies reporting data on AEs between the 2 groups, the treatment-related AEs did not differ significantly between combination treatment and PDE5 inhibitor monotherapy (odds ratio, 1.10; 95% CI, 0.66-1.85; *I*^2^ = 78%) (eAppendix 8 in the Supplement).

### Subgroups of Patients With ED

Participants with ED were further classified into those with lower urinary tract symptoms (LUTS), hypogonadism, prostatectomy-induced ED, or monotherapy-resistant ED or other subgroups. Other subgroups included monotherapy-naive individuals with diabetes, hypertension, or other primary ED with organic causes.

**Figure 2.**
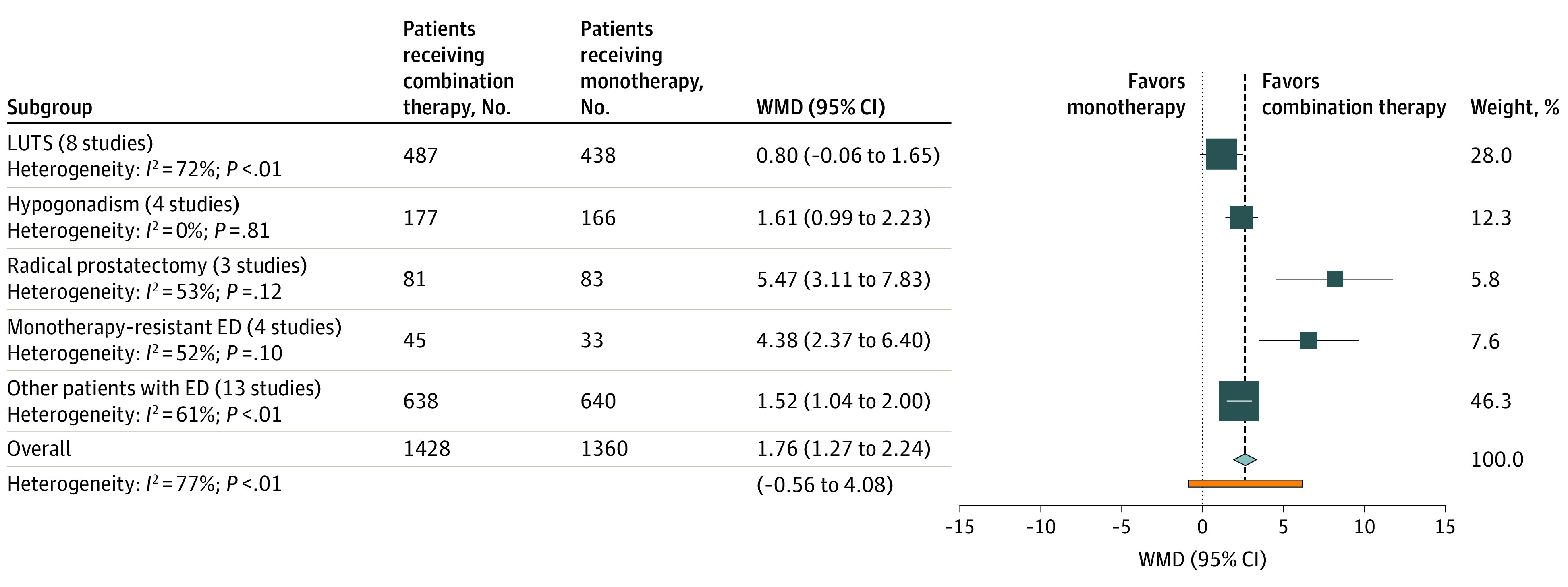
Forest Plot of the Mean Difference in International Index of Erectile Function (IIEF) Score With Combination Therapies vs Phosphodiesterase Type 5 inhibitors (PDE5i) Monotherapy in All Identified Subgroups ED indicates erectile dysfunction; LUTS, lower urinary tract symptoms; and WMD, weighted mean difference.

Compared with monotherapy, combination treatment was associated with a significant IIEF score improvement in patients with hypogonadism (WMD, 1.61; 95% CI, 0.99-2.23; *I*^2^ = 0%), with monotherapy-resistant ED (WMD, 4.38; 95% CI, 2.37-6.40; *I*^2^ = 52%), or with prostatectomy-induced ED (WMD, 5.47; 95% CI, 3.11-7.83; *I*^2^ = 53%) and those in other ED subgroups (WMD, 1.52; 95% CI, 1.04-2.00; *I*^2^ = 61%). Conversely, patients with LUTS, who were all treated with an α-blocker in addition to a PDE5 inhibitor, did not report a statistically significant change in erectile function. The outcomes of combination therapy compared with PDE5 inhibitor monotherapy in all identified subgroups are displayed in Figure 2 and eAppendix 9 in the Supplement. Furthermore, among all subgroups of patients with ED, the treatment-related AEs did not differ significantly between the combination therapy and monotherapy groups (eAppendix 10 in the Supplement).

To explore substantial heterogeneity, we performed subgroup and sensitivity analyses. Combination therapy was associated with a significant mean IIEF score improvement in both responders (WMD, 1.55; 95% CI, 1.06-2.04) and nonresponders (WMD, 3.02; 95% CI, 1.17-4.87) to PDE5 inhibitors (eAppendix 11 in the Supplement). Moreover, the significant IIEF score improvement associated with combination therapy of testosterone and antioxidants was maintained when we included only placebo-controlled RCTs or studies at a low risk of bias (eAppendix 12 and 13 in the Supplement).

### Grading Quality of Evidence

The quality of evidence was downgraded to moderate for the mean IIEF score change from baseline because of serious inconsistency (attributed to high levels of heterogeneity) and indirectness (attributed to various study selection criteria). For similar reasons, the quality of evidence for the number of AEs was also downgraded to moderate. Details about the grading of evidence for both outcomes are provided in eAppendix 14 in the Supplement.

## Discussion

This systematic review and meta-analysis demonstrated that combination therapy of 2 first-line ED treatments or other treatment modalities plus PDE5 inhibitors was associated with improved erectile function without increased treatment-related AEs, compared with PDE5 inhibitor monotherapy. Based on these findings, patients with resistant ED may experience good outcomes after receiving combination therapy without the risk of increased AEs. In patients undergoing radical prostatectomy, postoperative initiation of combination treatment may be associated with improved erectile function. The combination of PDE5 inhibitor with antioxidants, such as propionyl-l-carnitine or l-arginine, was associated with better outcomes compared with PDE5 inhibitors monotherapy. On the other hand, in patients with LUTS, α-blockers did not seem to be associated with greater advantages when coadministered with PDE5 inhibitors. Despite multiple subgroup and sensitivity analyses, the levels of heterogeneity remained high for all outcomes and the 95% PIs were wide for all measures, indicating high variability in the outcomes of different combination therapies in future studies.

These findings are not only statistically significant but also clinically important. In particular, the IIEF-ED score displays a minimal clinically important difference (MCID), defined as the smallest difference that patients may actually perceive as beneficial after treatment.^67^ To attain an MCID, a minimal IIEF-ED score improvement from baseline of 2 points for patients with mild ED, 5 points for moderate ED, and 7 points for severe ED must be reported.^67^ In the present study, because we compared the mean difference of both the IIEF-ED and IIEF-5 scores between combination therapy and PDE5 inhibitor monotherapy and not each group with its baseline IIEF score, we could not perform an MCID-based analysis. Still, the phenomenally modest additional mean improvement in IIEF score of 1.76 points after combination therapy compared with PDE5 inhibitor monotherapy could boost erectile function to an MCID in many patients. Based on the previous findings, in the 2 most difficult-to-treat subgroups (PDE5 inhibitor monotherapy–resistant ED and prostatectomy-induced ED), combination therapy displayed the best outcomes and led to an additional increase in IIEF score of more than 4 points compared with PDE5 inhibitor monotherapy.

Erectile dysfunction and LUTS are 2 of the most frequent conditions in aging male populations and thus have implications for quality of life.^68^ The direct association of α-blockers with cavernosal smooth muscle relaxation has been proven in both animals and humans.^69,70^ Moreover, it has been reported that the combination of α-blockers and PDE5 inhibitors leads to substantial tissue relaxation in the cavernosal and prostatic tissue.^70^ The findings in this study are in accordance with those of the most recent meta-analysis that reported no significant difference in the mean IIEF score change between combination therapy and PDE5 inhibitor monotherapy.^71^ Similarly, the nonbeneficial outcomes of pentoxifylline for erectile function was somewhat expected because pentoxifylline also presents controversial efficacy when used as a monotherapy for the management of ED.^72^ Therefore, pentoxifylline was not likely to further improve ED when administered in addition to PDE5 inhibitors.

Erectile dysfunction and hypogonadism often coexist in aging men, and androgens may also have a direct association with the corpora cavernosa.^11^ The latter led some researchers to evaluate erectile function improvement after combination therapy of testosterone and PDE5 inhibitors in patients with hypogonadism.^47,48,50^ The results of this study point toward an additive efficacy of combination treatment compared to monotherapy. Therefore, testosterone replacement therapy and PDE5 inhibitors may be preferred from the beginning of ED symptoms in patients with hypogonadism.^11^

This meta-analysis also highlighted the superiority of concomitant administration of substances with antioxidant properties (such as l-arginine or propionyl-l-carnitine) and PDE5 inhibitors. Given that PDE5 inhibitors improve nitric oxide (NO) bioavailability, increased oxidative stress may decrease the levels of NO and, in turn, may be associated with lower response rates to PDE5 inhibitors monotherapy.^73^ It has been reported that l-arginine increases the levels of NO^74^ and that propionyl-l-carnitine, through its antioxidant activity, decreases reactive oxygen species–mediated NO deactivation.^34^ Therefore, the concurrent administration of antioxidants with PDE5 inhibitors may represent an ED treatment that could improve the outcomes of PDE5 inhibitors. Still, further research into this treatment is necessary.

Recent data from a high-volume center demonstrated that, despite the advancements in surgical techniques and postoperative care, recovery from prostatectomy-induced ED has not substantially improved in the past decade, highlighting the need for novel treatment strategies.^75^ Findings from the present study suggest that, compared with monotherapy, combination treatment significantly improved erectile function in men who underwent radical prostatectomy. In this scope, combination therapeutic approaches could be a good solution for this difficult-to-treat subgroup of patients.

In the past few years, new ED treatment modalities, such as Li-ESWT, have been making their way through the clinical pipeline, and other treatments, such as platelet-rich plasma injections and stem cell therapy, have been gaining clinical attention.^76,77^ PDE5 inhibitors in combination with Li-ESWT seem to provide beneficial outcomes for nonresponders to PDE5 inhibitors.^78^ Accordingly, the superiority of platelet-rich plasma plus Li-ESWT over Li-ESWT monotherapy has been reported.^62^ Moreover, findings from an animal study have suggested that Li-ESWT combined with stem cell therapy is associated with improved neoangiogenesis and decreased penile corpora autophagy, compared with either treatment alone.^79^ All of these reports point toward these emerging therapies as potentially effective treatment modalities for the management of ED. Still, RCTs that compare different combination strategies are warranted to produce evidence for the optimal combination treatment.

### Limitations

This study has some limitations. First, we imputed missing SDs based on correlation coefficients reported in the included studies. Although this method is recommended by the Cochrane Collaboration and the robustness of the results was validated with a sensitivity analysis, the findings should be interpreted with caution. Moreover, given the scarcity of available data, we could not perform a subgroup analysis by severity of ED; therefore, firm conclusions about the association between improved outcomes and some combination treatment modalities in some patient subgroups should not be drawn. Second, we could not evaluate the long-term advantages of combination therapy because of the short follow-up period in all identified studies. Third, we did not identify any study that compared different types of combination treatments. The 95% PIs and the levels of heterogeneity remained high after subgroup and sensitivity analyses. High levels of heterogeneity were attributed to the different study design and selection criteria among the included studies. Accordingly, the potential publication bias also limited the extrapolation of these results.

## Conclusions

The findings of this systematic review and meta-analysis demonstrated that combination therapy was a safe and effective option for the management of ED in individuals who reported limited or no response after use of PDE5 inhibitors. Antioxidants added to PDE5 inhibitors was associated with improved ED without increasing the AEs, and the addition of daily tadalafil, Li-ESWT, or a vacuum erectile device seemed to be effective, but research data are scarce. Conversely, combination of PDE5 inhibitors and α-blockers was not associated with improved outcomes compared with PDE5 inhibitor monotherapy in patients with LUTS. These results suggest that combination therapy should be the initial preference in patients with hypogonadism or prostatectomy-induced ED. Nevertheless, substantial heterogeneity was detected across all analyses. The established therapeutic algorithms of ED should be reevaluated to consider combination therapy as the first-line treatment for refractory, complex, or difficult-to-treat cases of ED.

## Notes

Supplement. **eAppendix 1.** PubMed Search Syntax and Search String **eAppendix 2.** Flow Diagram of Study Selection Process **eAppendix 3.** References of All Excluded Studies With Reasons for Exclusion **eAppendix 4.** Risk of Bias in RCTs **eAppendix 5.** Risk of Bias in Non-RCTs **eAppendix 6.** Publication Bias Assessment With Funnel Plot and Egger’s Test **eAppendix 7.** Efficacy Based on Treatment Modality **eAppendix 8.** Adverse Events Based on Treatment Modality **eAppendix 9.** Efficacy Based on Impotent Subpopulation **eAppendix 10.** Adverse Events Based on Impotent Subpopulation **eAppendix 11.** Subgroup Analysis According to PDE5i Response **eAppendix 12.** Sensitivity Analysis Including Placebo-Controlled RCTs **eAppendix 13.** Sensitivity Analysis Including Studies at Low Risk of Bias **eAppendix 14.** Grading of Evidence
