# Supplementary material for: Assessment of Combination Therapies vs Monotherapy for Erectile Dysfunction: A Systematic Review and Meta-analysis
Source: JAMA Netw Open. 2021 Feb 18;4(2):e2036337. doi: 10.1001/jamanetworkopen.2020.36337 (PMC7893498; doi:10.1001/jamanetworkopen.2020.36337)
Supplement: Supplement. — eAppendix 1. PubMed Search Syntax and Search String eAppendix 2. Flow Diagram of Study Selection Process eAppendix 3. References of All Excluded Studies With Reasons for Exclusion eAppendix 4. Risk of Bias in RCTs eAppendix 5. Risk of Bias in Non-RCTs eAppendix 6. Publication Bias Assessment With Funnel Plot and Egger’s Test eAppendix 7. Efficacy Based on Treatment Modality eAppendix 8. Adverse Events Based on Treatment Modality eAppendix 9. Efficacy Based on Impotent Subpopulation eAppendix 10. Adverse Events Based on Impotent Subpopulation eAppendix 11. Subgroup Analysis According to PDE5i Response eAppendix 12. Sensitivity Analysis Including Placebo-Controlled RCTs eAppendix 13. Sensitivity Analysis Including Studies at Low Risk of Bias eAppendix 14. Grading of Evidence [file jamanetwopen-e2036337-s001.pdf]

Supplementary Online Content

Mykoniatis I, Pyrgidis N, Sokolakis I, et al. Assessment of combination therapies vs monotherapy for erectile dysfunction: a systematic review and meta-analysis. *JAMA Netw Open*. 2021;4(2):e2036337. doi:10.1001/jamanetworkopen.2020.36337

eAppendix 1. PubMed Search Syntax and Search String
eAppendix 2. Flow Diagram of Study Selection Process
eAppendix 3. References of All Excluded Studies With Reasons for Exclusion
eAppendix 4. Risk of Bias in RCTs
eAppendix 5. Risk of Bias in Non-RCTs
eAppendix 6. Publication Bias Assessment With Funnel Plot and Egger's Test
eAppendix 7. Efficacy Based on Treatment Modality
eAppendix 8. Adverse Events Based on Treatment Modality
eAppendix 9. Efficacy Based on Impotent Subpopulation
eAppendix 10. Adverse Events Based on Impotent Subpopulation
eAppendix 11. Subgroup Analysis According to PDE5i Response
eAppendix 12. Sensitivity Analysis Including Placebo-Controlled RCTs
eAppendix 13. Sensitivity Analysis Including Studies at Low Risk of Bias
eAppendix 14. Grading of Evidence

This supplementary material has been provided by the authors to give readers additional information about their work.

©2021 Mykoniatis I et al. *JAMA Network Open*

eAppendix 1. PubMed Search Syntax and Search String

Search syntax

ID	Search
#1	"Sexual dysfunction" [All fields]
#2	"Sexual disorder" [All fields]
#3	"Erectile function" [All fields]
#4	Erection [All fields]
#5	"Erectile dysfunction" [All fields]
#6	"Erectile disorder" [All fields]
#7	Impotence [All fields]
#8	"International index of erectile function" [All fields]
#9	IIEF [All fields]
#10	"penile erection" [MeSH Terms]
#11	OR #1-11
#12	“combination treatment” [All fields]
#13	“combination therapy” [All fields]
#14	“multimodal therapy” [All fields]
#15	“multimodal treatment” [All fields]
#16	“combined treatment” [All fields]
#17	“combined therapy” [All fields]
#18	“add on” [All fields]
#19	add-on [All fields]
#20	co-treatment [All fields]
#21	cotreatment [All fields]
#22	coadministration [All fields]
#23	co-administration [All fields]
#24	coadmin* [All fields]
#25	cotreat* [All fields]
#26	synergistic [All fields]
#27	synerg* [All fields]
#28	OR #11-27
#29	#11 AND #28

Search string

(((((((("sexual dysfunction"[All Fields] OR "sexual disorder"[All Fields]) OR "erectile dysfunction"[All Fields]) OR "erectile disorder"[All Fields]) OR "impotence"[All Fields]) OR "erectile function"[All Fields]) OR "erection"[All Fields]) OR "international index of erectile function"[All Fields]) OR "IIEF"[All Fields]) OR "penile erection"[MeSH Terms]) AND ((((((((((((((("combination group"[All Fields] OR "combination treatment"[All Fields]) OR "combination therapy"[All Fields]) OR "multimodal therapy"[All Fields]) OR "multimodal treatment"[All Fields]) OR "combined treatment"[All Fields]) OR "combined therapy"[All Fields]) OR "add-on"[All Fields]) OR "add-on"[All Fields]) OR "co-treatment"[All Fields]) OR "cotreatment"[All Fields]) OR "coadministration"[All Fields]) OR "co-administration"[All Fields]) OR "coadmin*"[All Fields]) OR "cotreat*"[All Fields]) OR "synergistic"[All Fields]) OR "synerg*"[All Fields])

The search strategy was developed for PubMed and modified accordingly for the other databases.

eAppendix 2. Flow Diagram of Study Selection Process

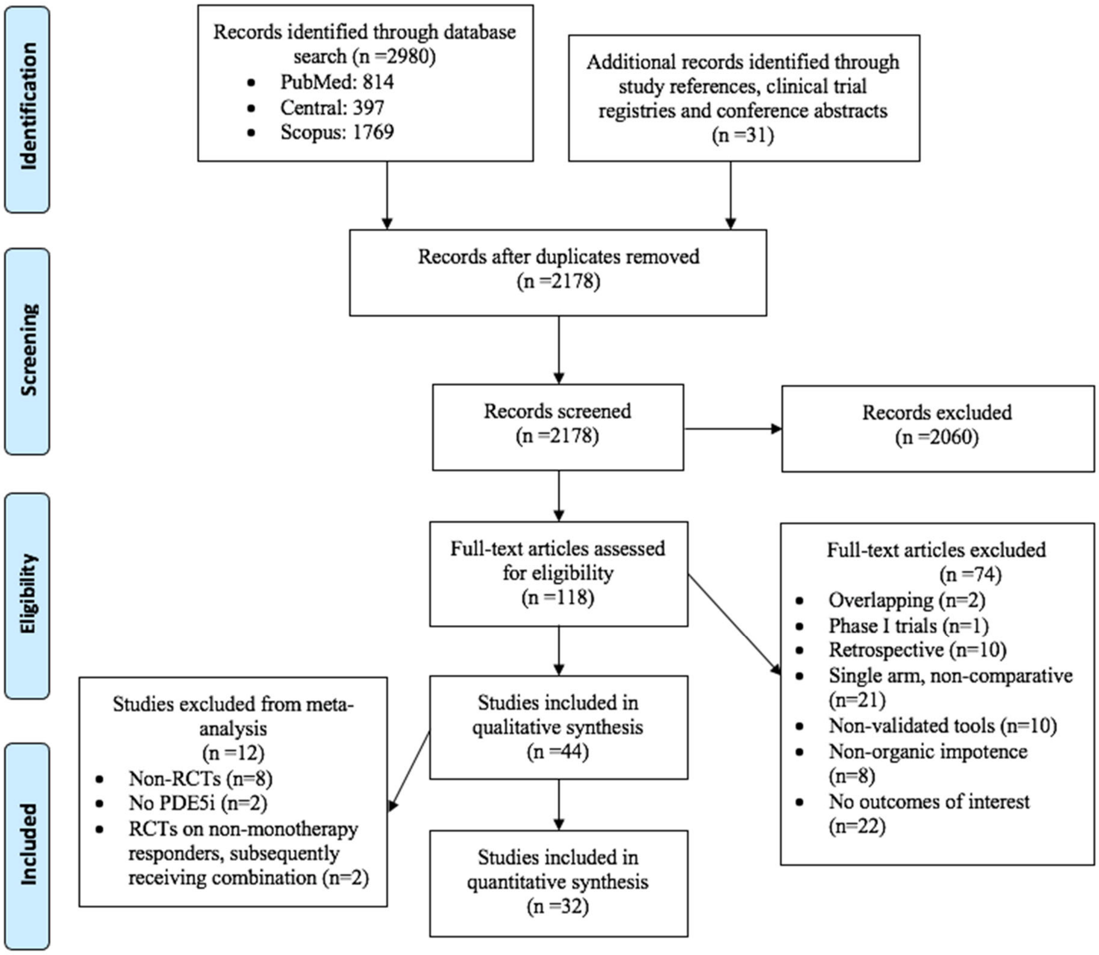

PDE5i: phosphodiesterase type 5 inhibitor; RCT: randomized controlled trial.

©2021 Mykoniatis I et al. *JAMA Network Open*

eAppendix 3. References of All Excluded Studies With Reasons for Exclusion

Overlapping study records

1. Ruffo A, Franco M, Illiano E, Stanojevic N. Effectiveness and safety of Platelet rich Plasma (PrP)cavernosal injections plus external shock wave treatment for penile erectile dysfunction: first results from a prospective, randomized, controlled, interventional study. Eur Urol Suppl 2019;18(1):e1622-e1623.
2. Sebastianelli A, Spatafora P, Frizzi J, et al. Which Drug to Discontinue 3 Months After Combination Therapy of Tadalafil plus Tamsulosin for Men with Lower Urinary Tract Symptom and Erectile Dysfunction? Results of a Prospective Observational Trial. Eur Urol Focus 2019;

Phase I clinical trials

1. Diamond LE, Earle DC, Garcia WD, Spana C. Co-administration of low doses of intranasal PT-141, a melanocortin receptor agonist, and sildenafil to men with erectile dysfunction results in an enhanced erectile response. Urology 2005;65(4 CC-Common Mental Disorders CC-Urology):755-759.

Retrospective observational trials

1. Giuliano F, Joussain C, Denys P. Safety and Efficacy of Intracavernosal Injections of AbobotulinumtoxinA (Dysport®) as Add on Therapy to Phosphosdiesterase Type 5 Inhibitors or Prostaglandin E1 for Erectile Dysfunction-Case Studies. Toxins (Basel) 2019;11(5).
2. Kimura M, Caso JR, Bañez LL, et al. Predicting participation in and successful outcome of a penile rehabilitation programme using a phosphodiesterase type 5 inhibitor with a vacuum erection device after radical prostatectomy. BJU Int 2012;110(11 Pt C):E931-8.
3. Labairu-Huerta L, Padilla-Fernández B, Arrondo-Arrondo JL, et al. PDE-5 inhibitors in monotherapy versus combination therapy in a sample of 1200 patients with erectile dysfunction. Arch Ital di Urol Androl organo Uff [di] Soc Ital di Ecogr Urol e Nefrol 2015;87(3):204–9.
4. Mydlo JH, Volpe MA, Macchia RJ. Initial results utilizing combination therapy for patients with a suboptimal response to either alprostadil or sildenafil monotherapy. Eur Urol 2000;38(1):30–4.
5. Mydlo JH, Volpe MA, MacChia RJ. Results from different patient populations using combined therapy with alprostadil and sildenafil: predictors of satisfaction. BJU Int 2000;86(4):469–73.
6. Mydlo JH, Viterbo R, Crispen P. Use of combined intracorporal injection and a phosphodiesterase-5 inhibitor therapy for men with a suboptimal response to sildenafil and/or vardenafil monotherapy after radical retropubic prostatectomy. BJU Int 2005;95(6):843–6.
7. Raina R, Nandipati KC, Agarwal A, Mansour D, Kaelber DC, Zippe CD. Combination therapy: medicated urethral system for erection enhances sexual satisfaction in sildenafil citrate failure following nerve-sparing radical prostatectomy. J Androl 2005;26(6):757–60.
8. Raina R, Pahlajani G, Agarwal A, Jones S, Zippe C. Long-term potency after early use of a vacuum erection device following radical prostatectomy. BJU Int 2010;106(11):1719–22.

9. Verze P, Capece M, Creta M, et al. Efficacy and safety of low-intensity shockwave therapy plus tadalafil 5 mg once daily in men with type 2 diabetes mellitus and erectile dysfunction: a matched-pair comparison study. Asian J Androl 2020;22(4):379.
10. Yassin AA, Saad F, Diede HE. Testosterone and erectile function in hypogonadal men unresponsive to tadalafil: results from an open-label uncontrolled study. Andrologia 2006;38(2 CC-Urology):61-68.

Single arm, non-comparative studies

1. Bang WJ, Oh CY, Yoo C, et al. Efficacy and safety of the simultaneous administration of mirodenafil and an α-blocker in men with BPH-LUTS: A multicenter open-label prospective study. Int J Impot Res 2013;25(4):149–54.
2. Bechara A, Casabé A, De Bonis W, Nazar J. Efectividad de las ondas de choque de baja intensidad (LIWS) en varones con disfunción eréctil (DE) no respondedores a IPDE5. Estudio piloto. Arch Españoles Urol 2015;68(2):152–60.
3. Canguven O, Bailen J, Fredriksson W, Bock D, Burnett AL. Combination of vacuum erection device and PDE5 inhibitors as salvage therapy in PDE5 inhibitor nonresponders with erectile dysfunction. J Sex Med 2009;6(9):2561–7.
4. Casabé A, Roehrborn CG, Da Pozzo LF, et al. Efficacy and safety of the coadministration of tadalafil once daily with finasteride for 6 months in men with lower urinary tract symptoms and prostatic enlargement secondary to benign prostatic hyperplasia. J Urol 2014;191(3):727–33.
5. Chatterjee R, Kottaridis PD, McGarrigle HH, Linch DC. Management of erectile dysfunction by combination therapy with testosterone and sildenafil in recipients of high-dose therapy for haematological malignancies. Bone Marrow Transplant 2002;29(7):607–10.
6. Chung BH, Lee JY, Lee SH, Yoo SJ, Lee SW, Oh CY. Safety and efficacy of the simultaneous administration of udenafil and an α-blocker in men with erectile dysfunction concomitant with BPH/LUTS. Int J Impot Res 2009;21(2):122–8.
7. Gacci M, Vittori G, Tosi N, et al. A Randomized, Placebo-Controlled Study to Assess Safety and Efficacy of Vardenafil 10mg and Tamsulosin 0.4mg vs. Tamsulosin 0.4mg Alone in the Treatment of Lower Urinary Tract Symptoms Secondary to Benign Prostatic Hyperplasia. J Sex Med 2012;9(6):1624–33.
8. Garcia JA, Sanchez PE, Fraile C, Escovar P. Testosterone undecanoate improves erectile dysfunction in hypogonadal men with the metabolic syndrome refractory to treatment with phosphodiesterase type 5 inhibitors alone. Andrologia 2011;43(5):293–6.
9. Gholamine B, Shafiei M, Motevallian M, Mahmoudian M. Effects of pioglitazone on erectile dysfunction in sildenafil poor-responders: a randomized, controlled study. J Pharm Pharm Sci a Publ Can Soc Pharm Sci Soc Can des Sci Pharm 2008;11(1):22–31.
10. Greenstein A, Mabjeesh NJ, Sofer M, Kaver I, Matzkin H, Chen J. Does sildenafil combined with testosterone gel improve erectile dysfunction in hypogonadal men in whom testosterone supplement therapy alone failed? J Urol 2005;173(2):530–2.
11. Kalinchenko SY, Kozlov GI, Gontcharov NP, Katsiya G V. Oral testosterone undecanoate reverses erectile dysfunction associated with diabetes mellitus in patients failing on sildenafil citrate therapy alone. Aging Male 2003;6(2):94–9.
12. Kaplan SA, Reis RB, Kohn IJ, Shabsigh R, Te AE. Combination therapy using oral alpha-blockers and intracavernosal injection in men with erectile dysfunction. Urology 1998;52(5):739–43.
13. Kondoh N, Higuchi Y, Maruyama T, Nojima M, Yamamoto S, Shima H. Salvage

therapy trial for erectile dysfunction using phosphodiesterase type 5 inhibitors and vitamin E: Preliminary report. Aging Male 2008;11(4):167–70.
14. Lee JY, Cho SY, Oh CY, et al. Efficacy and safety of combination therapy with mirodenafil and α 1-blocker for benign prostatic hyperplasia-induced lower urinary tract symptoms accompanied by erectile dysfunction: A multicenter, open-label, prospective study. Int J Impot Res 2011;23(6):249–56.
15. Özkıdık M, Gökce Mİ, Yaman Ö. Efficacy of tadalafil treatment on erectile dysfunction in patients under dutasteride treatment: A prospective non-randomized comparative study. Turkish J Urol 2018;44(4):294–7.
16. Park MG, Yeo JK, Cho D-YY, et al. The efficacy of combination treatment with injectable testosterone undecanoate and daily tadalafil for erectile dysfunction with testosterone deficiency syndrome. J Sex Med 2015;12(4):966-974.
17. Tas A, Ersoy A, Ersoy C, Gullulu M, Yurtkuran M. Efficacy of sildenafil in male dialysis patients with erectile dysfunction unresponsive to erythropoietin and/or testosterone treatments. Int J Impot Res 2006;18(1):61–8.
18. Tsai C-C, Wang C-J, Lee Y-C, et al. Low-Intensity Extracorporeal Shockwave Therapy Can Improve Erectile Function in Patients Who Failed to Respond to Phosphodiesterase Type 5 Inhibitors. Am J Mens Health 2017;11(6):1781–90.
19. Tuken M, Culha MG, Serefoglu EC. Efficacy and safety of dapoxetine/sildenafil combination tablets in the treatment of men with premature ejaculation and concomitant erectile dysfunction-DAP-SPEED Study. Int J Impot Res 2019;31(2):92–6.
20. Yang L, Cai J, He D. Linear focus extracorporeal shockwave therapy in the treatment of erectile dysfunction: a multi-center, prospective, randomized, double-blinded, placebo controlled study. J Endourol 2017;31:A330-.
21. Yassin D-J, Yassin AA, Hammerer PG. Combined testosterone and vardenafil treatment for restoring erectile function in hypogonadal patients who failed to respond to testosterone therapy alone. J Sex Med 2014;11(2):543–52.

Studies evaluating combination therapy versus monotherapy with non-validated tools

1. Giuliano F, Joussain C, Denys P. Safety and Efficacy of Intracavernosal Injections of AbobotulinumtoxinA (Dysport®) as Add on Therapy to Phosphosdiesterase Type 5 Inhibitors or Prostaglandin E1 for Erectile Dysfunction-Case Studies. Toxins (Basel) 2019;11(5).
2. Kimura M, Caso JR, Bañez LL, et al. Predicting participation in and successful outcome of a penile rehabilitation programme using a phosphodiesterase type 5 inhibitor with a vacuum erection device after radical prostatectomy. BJU Int 2012;110(11 Pt C):E931-8.
3. Labairu-Huerta L, Padilla-Fernández B, Arrondo-Arrondo JL, et al. PDE-5 inhibitors in monotherapy versus combination therapy in a sample of 1200 patients with erectile dysfunction. Arch Ital di Urol Androl organo Uff [di] Soc Ital di Ecogr Urol e Nefrol 2015;87(3):204–9.
4. Mydlo JH, Volpe MA, Macchia RJ. Initial results utilizing combination therapy for patients with a suboptimal response to either alprostadil or sildenafil monotherapy. Eur Urol 2000;38(1):30–4.
5. Mydlo JH, Volpe MA, MacChia RJ. Results from different patient populations using combined therapy with alprostadil and sildenafil: predictors of satisfaction. BJU Int 2000;86(4):469–73.
6. Mydlo JH, Viterbo R, Crispen P. Use of combined intracorporal injection and a phosphodiesterase-5 inhibitor therapy for men with a suboptimal response to sildenafil

and/or vardenafil monotherapy after radical retropubic prostatectomy. BJU Int 2005;95(6):843–6.
7. Raina R, Nandipati KC, Agarwal A, Mansour D, Kaelber DC, Zippe CD. Combination therapy: medicated urethral system for erection enhances sexual satisfaction in sildenafil citrate failure following nerve-sparing radical prostatectomy. J Androl 2005;26(6):757–60.
8. Raina R, Pahlajani G, Agarwal A, Jones S, Zippe C. Long-term potency after early use of a vacuum erection device following radical prostatectomy. BJU Int 2010;106(11):1719–22.
9. Verze P, Capece M, Creta M, et al. Efficacy and safety of low-intensity shockwave therapy plus tadalafil 5 mg once daily in men with type 2 diabetes mellitus and erectile dysfunction: a matched-pair comparison study. Asian J Androl 2020;22(4):379.
10. Yassin AA, Saad F, Diede HE. Testosterone and erectile function in hypogonadal men unresponsive to tadalafil: results from an open-label uncontrolled study. Andrologia 2006;38(2 CC-Urology):61-68.

Studies in potent participants or in patients with non-organic ED

1. Alwan AA, Ajeel HT, Abd AH, et al. A comparative study for the effectiveness of tamsulosin alone versus tamsulosin plus tadalafil combination as an expulsive medical treatment in the management of lower ureteric calculous in al-diwaniyah teaching hospital. Int J Res Pharm Sci 2019;10(2):1551-1555.
2. Cantoro U, Catanzariti F, Lacetera V, Quaresima L, Muzzonigro G, Polito M. Comparison of tamsulosin vs tamsulosin/sildenafil effectiveness in the treatment of erectile dysfunction in patients affected by type III chronic prostatitis. Arch Ital di Urol Androl organo Uff [di] Soc Ital di Ecogr Urol e Nefrol 2013;85(3):109–12.
3. De Rose AF, Giglio M, Traverso P, Lantieri P, Carmignani G. Combined oral therapy with sildenafil and doxazosin for the treament of non-organic erectile dysfunction refractory to sildenafil monotherapy. Int J Impot Res 2002;14(1):50–3.
4. Ibishev KS. Efficiency of combined therapy with impaza and type 5 phosphodiesterase inhibitors in prophylactics of posttraumatic erectile dysfunction. Bull Exp Biol Med 2009;148(2):322–4.
5. Kosilov K V, Kuzina IG, Kuznetsov V, Kosilova EK. Improvement of the symptoms of lower urinary tract and sexual dysfunction with tadalafil and solifenacin after the treatment of benign prostatic hyperplasia with dutasteride. Prostate Int 2020;
6. Kumar S, Kondareddy C, Ganesamoni R, Nanjappa B, Singh SK. Randomized controlled trial to assess the efficacy of the combination therapy of alfuzosin and tadalafil in patients with lower urinary tract symptoms due to benign prostatic hyperplasia. LUTS Low Urin tract symptoms 2014;6(1):35-40.
7. Singh DV, Mete UK, Mandal AK, Singh SK. A comparative randomized prospective study to evaluate efficacy and safety of combination of tamsulosin and tadalafil vs. tamsulosin or tadalafil alone in patients with lower urinary tract symptoms due to benign prostatic hyperplasia. J Sex Med 2014;11(1):187–96.
8. Tak G, Singh A, Ganpule A, Sabnis R, Desai M. Double blind, randomized, placebo controlled study of Tadalafil with Dutasteride vs Tadalafil with placebo for Lower urinary tracts symptoms secondary to benign prostatic hyperplasia: a single centre study. Child Dev 2019;201(4):e462-.

Studies not reporting clinical outcomes of interest

1. Akhvlediani ND, Matyukhov IP. [New data on the effectiveness of Tadalafil alone and in combination with NeyroDoz in treating erectile dysfunction associated with

secondary premature ejaculation]. Urologiia 2016;(5):52–6.
2. Aksonov P, Gorpynchenko I, Romaniuk M. Assessment of the treatment efficacy of the low-energy shock wave therapy in patients with vascular erectile dysfunction. J Sex Med Conf 18th Congr Eur Soc Sex Med Madrid spain Conf start 20160204 Conf end 20160206 Conf Publ 2016;13(5 SUPPL. 2):S115.
3. Aleksandre B, Andro D, Milad H, Nino A, George B. Combination treatment with low intensity extracorporeal shock waves and PDE5 inhibitors for vasculogenic erectile dysfunction: a comparative study. Eur Urol Suppl 2019;18(12):e3628–9.
4. Aliaev I, Vinarov AZ, Akhvlediani ND. Choice of treatment of erectile dysfunction associated with hypogonadism. Urologiia 2010;(4 CC-Urology):37-8, 40-2.
5. Dmitriev DG, Gamidov SI, Mazo EB, Ovchinnikov RI. A new approach to raising efficiency of pharmacotherapy of erectile dysfunction. Ter Arkh 2002;74(10):75–7.
6. Kamalov AA, Khodyreva LA, Dudareva AA, et al. [Results of a multicenter non-interventional study on the efficacy and safety of NeyroDoz complex for sexual dysfunction in men]. Urologiia 2016;(1 Suppl 1):47–53.
7. Kamalov AA, Nizov AN. [Efficiency of phosphodiesterase-5 inhibitors for treatment of lower urinary tract symptoms in patients with benign prostatic hyperplasia and concomitant erectile dysfunction]. Urologiia 2019;(4):130–4.
8. Kamalov AA, Osmolovskiĭ BE, Okhobotov DA, et al. [Combined treatment of patients with erectile dysfunction and urination disorders]. Urologiia 2013;(3):29-30,32-33.
9. Kamalov AA, Takhirzade AM. [Approaches to medical management of patients with high risk of progressing of benign prostatic hyperplasia depending on concomitant erectile dysfunction]. Urologiia 2018;(3):70–7.
10. Karpukhin I V, Bogomol'nyĭ VA. [Physical factors in the treatment and rehabilitation of patients with chronic prostatitis complicated by impotence]. Vopr Kurortol Fizioter Lech Fiz Kult 1999;(2):25–7.
11. Kim DY, Park YJ, Sung LH, Kim JC. Efficacy and safety of combination of alfuzosin and low dose udenafil once daily versus monotherapy in patients with comorbid lower urinary tract symptoms and erectile dysfunction: randomized prospective open-label study. Eur Urol Suppl 2012;11(1):e754-754a.
12. Kyzlasov PS, Plekhanova OA, Volodin DI, et al. New approach to preserve male sexual function after nerve-sparing radical prostatectomy. Eur Urol Suppl 2019;18(12):e2063-.
13. Li GY, Liang JH, Meng ZB, et al. Low-dose testosterone undecanoate capsules combined with tadalafil for late-onset hypogonadism accompanied with ED. Zhonghua nan ke xue [National J andrology] 2013;19(7):630-633.
14. Lombardo F, Tsamatropoulos P, Piroli E, et al. Treatment of erectile dysfunction due to C677T mutation of the MTHFR gene with vitamin B6 and folic acid in patients non responders to PDE5i. J Sex Med 2010;7(1 Pt 1):216–23.
15. Mazo EB, Dmitriev DG, Gamidov SI, Ovchinnikov RI. [Sildenafil and alprostadil in the combined drug therapy of erectile dysfunction]. Urologiia 2002;(3):39–43.
16. Mazo EB, Gamidov SI, Iremashvili V V, Sotnikova EM. [The role of testosterone drugs in combined therapy of erectile dysfunction in patients with metabolic syndrome]. Urologiia 2007;(4):63-66,69.
17. Mazo EB, Gamidov SI, Sotnikova EM. Effects of different treatments on endothelial function in patients with erectile dysfunction and hypogonadism]. [Russian. Ter Arkh 2008;80(12 CC-Urology):59-63.
18. Piubello G. Efficacy of a nutraceutical preparation as addon treatment in patients with erectile dysfunction treated with 5-PDE inhibitors: A pilot study. J Andrological Sci 2010;17(4):178–82.

19. Ridha M, Renaldo J, Budiono, Hardjowijoto S. Randomised controlled trial of tamsulosin and tadalafil for lower urinary tract symptoms with erectile dysfunction. BJU Int 2014;114:1-2.
20. Tiequn L. Study on relevance of the medical treatments between ED and LUTS. Chinese J Androl 2008;22(12):26–9.
21. Volkov AA, Petrichko MI, Budnik N V, Dukhin AR. [Tadalafil in patients with benign prostatic hyperplasia during conservative combined therapy]. Urologiia 2013;(2):56,58-59.
22. Xin Z-C. Safety and efficacy of sildenafil and doxazosin gits for treating chinese patients with ED with BPH/LUTS. J Sex Med 2012;9:125.

©2021 Mykoniatis I et al. *JAMA Network Open*

eAppendix 4. Risk of Bias in RCTs

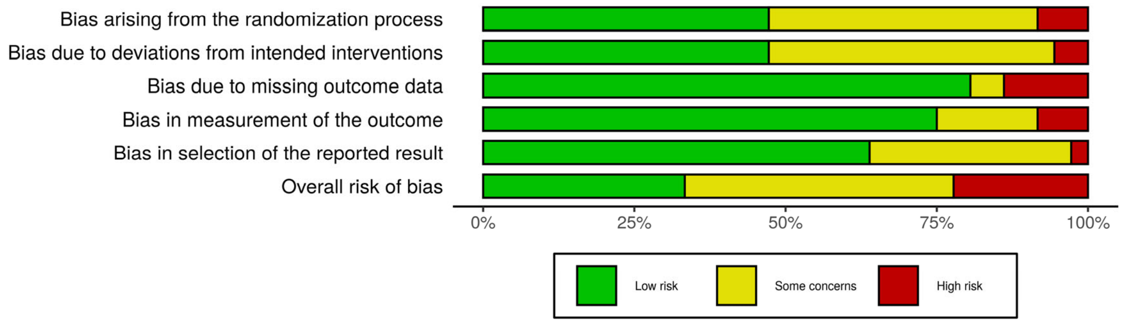

©2021 Mykoniatis I et al. *JAMA Network Open*

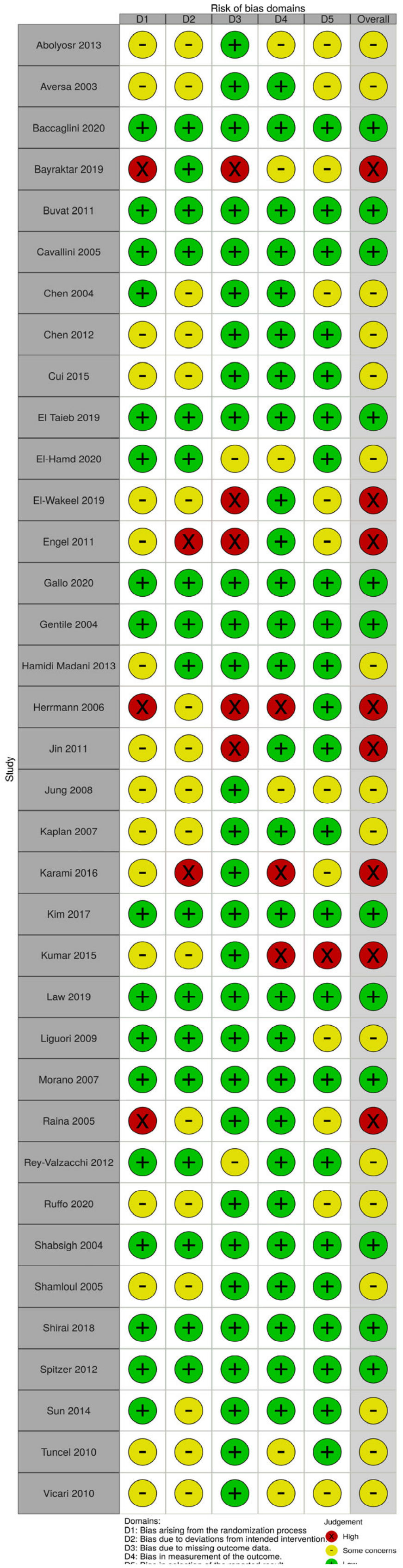

©2021 Mykoniatis I et al. *JAMA Network Open*

eAppendix 5. Risk of Bias in Non-RCTs

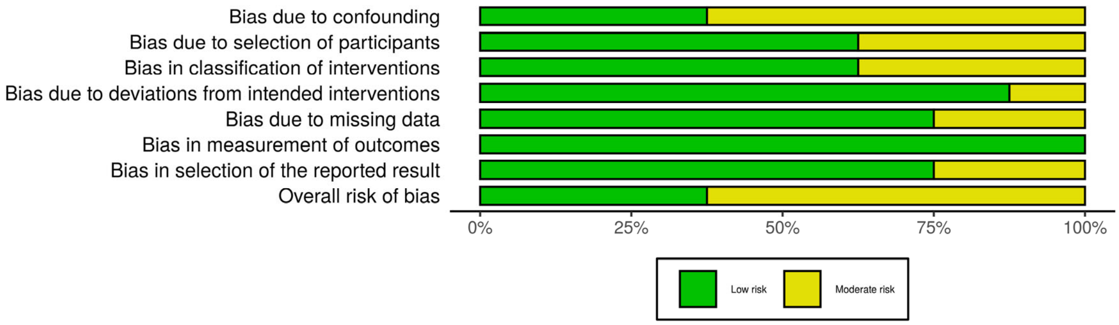

©2021 Mykoniatis I et al. *JAMA Network Open*

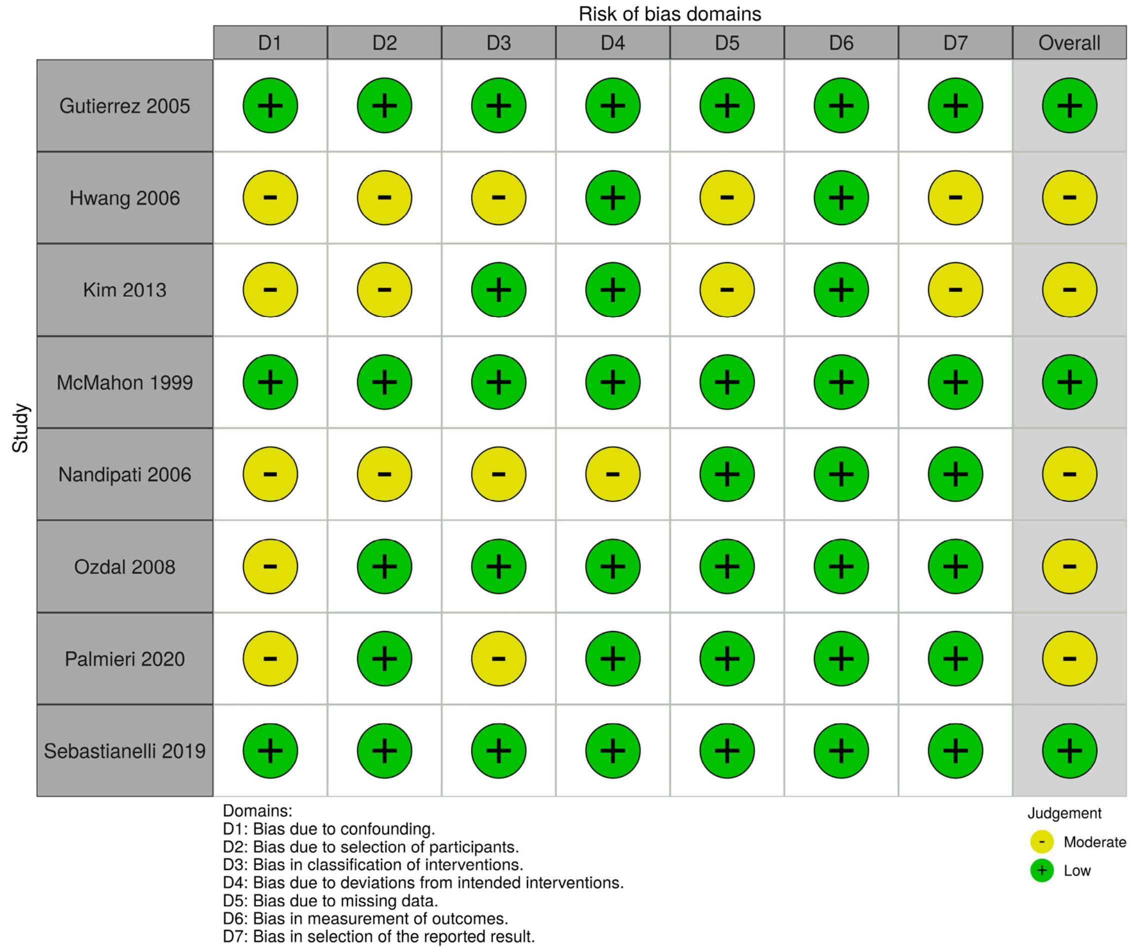

©2021 Mykoniatis I et al. *JAMA Network Open*

eAppendix 6. Publication Bias Assessment With Funnel Plot and Egger's Test

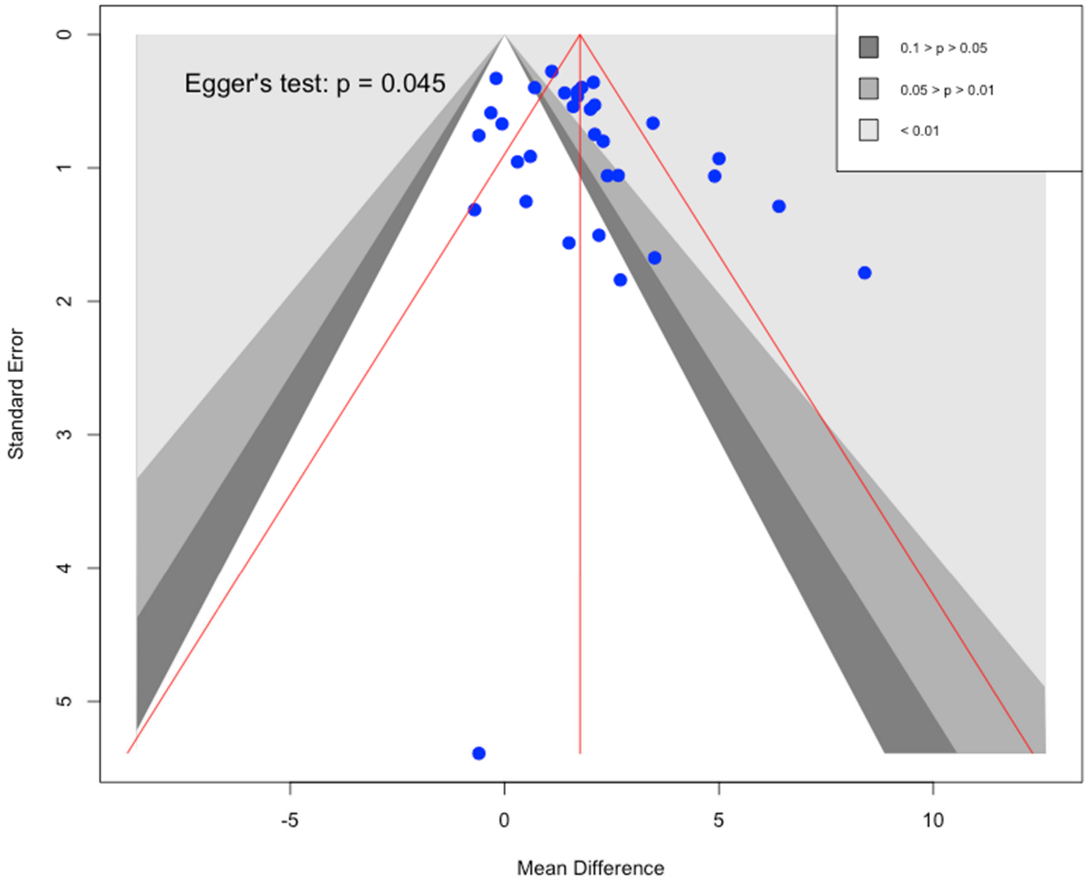

eAppendix 7. Efficacy Based on Treatment Modality

Study	Combination therapy Patients	MD	SD	Monotherapy Patients	MD	SD	WMD	95% CI	Weight
**PDE5i + A-blocker versus PDE5i**									
Abolyosr 2013	50	4.6	3.38	50	4.6	3.32	-0.06	[-1.37; 1.25]	3.7%
Jin 2011	137	6.9	3.53	66	4.8	3.53	2.10	[1.06; 3.14]	4.1%
Jung 2008	49	10.4	4.02	52	8.1	4.02	2.30	[0.73; 3.87]	3.3%
Kaplan 2007	18	9.5	2.95	19	7.1	3.48	2.40	[0.33; 4.47]	2.6%
Karami 2016	58	7.6	2.10	60	7.8	1.38	-0.20	[-0.84; 0.44]	4.6%
Kim 2017	134	9.2	4.98	152	9.5	4.93	-0.32	[-1.47; 0.83]	3.9%
Liguori 2009	21	5.3	2.89	19	4.7	2.88	0.60	[-1.19; 2.39]	3.0%
Tuncel 2010	20	7.5	3.02	20	7.2	3.02	0.30	[-1.57; 2.17]	2.9%
Heterogeneity: I^2 = 72%, p < 0.01	**487**			**438**			**0.80**	**[-0.06; 1.65]**	**28.0%**
**PDE5i + Testosterone versus PDE5i**									
Buvat 2011	72	3.6	7.40	66	3.1	7.30	0.50	[-1.95; 2.95]	2.2%
Aversa 2003	10	7.4	2.88	10	1.0	2.88	6.40	[3.88; 8.92]	2.1%
Shabsigh 2004	37	4.7	1.90	33	3.1	2.53	1.60	[0.54; 2.66]	4.0%
Shamloul 2005	10	2.2	1.09	10	0.5	0.80	1.70	[0.86; 2.54]	4.4%
Spitzer 2012	58	2.0	8.07	57	-0.2	8.07	2.20	[-0.75; 5.15]	1.8%
Heterogeneity: I^2 = 71%, p < 0.01	**187**			**176**			**2.27**	**[0.90; 3.65]**	**14.5%**
**PDE5i + LiESWT versus PDE5i**									
Baccaglini 2020	36	-8.9	7.33	41	-12.4	7.33	3.50	[0.22; 6.78]	1.5%
Heterogeneity: I^2 = NA%, p = NA	**36**			**41**			**3.50**	**[0.22; 6.78]**	**1.5%**
**PDE5i + Aspirin versus PDE5i**									
Bayraktar 2019	67	7.5	4.40	65	8.1	4.30	-0.60	[-2.08; 0.88]	3.4%
Heterogeneity: I^2 = NA%, p = NA	**67**			**65**			**-0.60**	**[-2.08; 0.88]**	**3.4%**
**PDE5i + Antioxidant versus PDE5i**									
Cavallini 2005	32	14.8	3.25	35	9.8	4.33	5.00	[3.18; 6.82]	2.9%
El Taieb 2019	27	9.8	2.75	27	7.7	2.75	2.10	[0.63; 3.57]	3.4%
El-Hamd 2020	30	13.7	1.86	30	12.3	1.53	1.40	[0.54; 2.26]	4.3%
El-Wakeel 2019	30	4.8	1.47	29	3.0	1.58	1.80	[1.02; 2.58]	4.4%
Gallo 2020	100	7.1	1.95	99	6.0	1.95	1.10	[0.56; 1.64]	4.7%
Gentile 2004	20	2.8	1.77	20	0.8	1.77	2.00	[0.90; 3.10]	4.0%
Morano 2007	8	8.7	3.84	8	6.0	3.51	2.70	[-0.91; 6.31]	1.3%
Shirai 2018	13	2.6	2.05	13	-0.0	3.21	2.65	[0.58; 4.72]	2.6%
Vicari 2010	53	5.4	8.07	53	3.9	8.01	1.50	[-1.56; 4.56]	1.7%
Heterogeneity: I^2 = 59%, p = 0.01	**313**			**314**			**1.99**	**[1.34; 2.63]**	**29.4%**
**PDE5i + ACEi versus PDE5i**									
Chen 2012	31	8.7	1.45	31	6.6	1.37	2.07	[1.37; 2.77]	4.5%
Heterogeneity: I^2 = NA%, p = NA	**31**			**31**			**2.07**	**[1.37; 2.77]**	**4.5%**
**PDE5i + PDE5i versus PDE5i**									
Cui 2015	90	9.5	3.08	90	7.8	3.14	1.70	[0.79; 2.61]	4.3%
Heterogeneity: I^2 = NA%, p = NA	**90**			**90**			**1.70**	**[0.79; 2.61]**	**4.3%**
**PDE5i + VED versus PDE5i**									
Engel 2011	13	17.7	2.87	7	9.3	4.23	8.40	[4.90; 11.90]	1.4%
Heterogeneity: I^2 = NA%, p = NA	**13**			**7**			**8.40**	**[4.90; 11.90]**	**1.4%**
**PDE5i + Folic acid versus PDE5i**									
Hamidi Madani 2013	35	5.1	3.84	48	1.7	0.99	3.46	[2.16; 4.76]	3.7%
Heterogeneity: I^2 = NA%, p = NA	**35**			**48**			**3.46**	**[2.16; 4.76]**	**3.7%**
**PDE5i + Atorvastatin versus PDE5i**									
Herrmann 2006	8	7.7	6.45	4	8.3	9.76	-0.60	[-11.16; 9.96]	0.2%
Heterogeneity: I^2 = NA%, p = NA	**8**			**4**			**-0.60**	**[-11.16; 9.96]**	**0.2%**
**PDE5i + Pentoxifylline versus PDE5i**									
Kumar 2015	120	9.7	3.11	117	9.0	3.03	0.70	[-0.08; 1.48]	4.4%
Law 2019	27	1.8	4.63	23	2.5	4.63	-0.70	[-3.27; 1.87]	2.1%
Heterogeneity: I^2 = 4%, p = 0.31	**147**			**140**			**0.56**	**[-0.26; 1.38]**	**6.5%**
**PDE5i + Metformin versus PDE5i**									
Rey-Valzacchi 2012	14	5.5	0.10	6	0.6	2.60	4.90	[2.82; 6.98]	2.6%
Heterogeneity: I^2 = NA%, p = NA	**14**			**6**			**4.90**	**[2.82; 6.98]**	**2.6%**
Heterogeneity: I^2 = 77%, p < 0.01	**1428**			**1360**			**1.76**	**[1.27; 2.24]**	**100.0%**
**Prediction interval**								**[-0.56; 4.08]**	

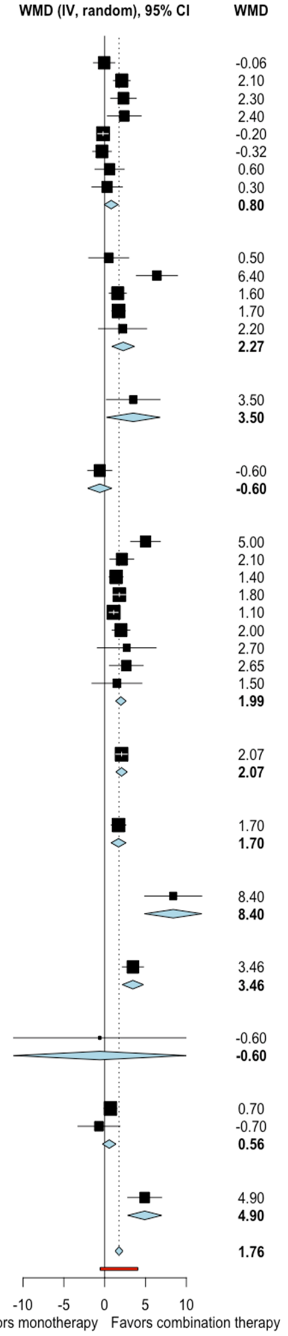

Mean difference in IIEF score with combination therapy versus monotherapy

Forest plot of the mean difference in IIEF score regarding different combination therapies versus PDE5i monotherapy with single study estimates. ACEi: angiotensin converting enzyme inhibitor; CI: confidence interval; ED: erectile dysfunction; IIEF: international index of erectile function; IV: inverse variance; Li-ESWT: low-intensity extracorporeal shockwave therapy; MD: mean difference; PDE5i: phosphodiesterase type 5 inhibitors; SD: standard deviation; VED: vacuum erectile device; WMD: weighted mean difference.

©2021 Mykoniatis I et al. *JAMA Network Open*

eAppendix 8. Adverse Events Based on Treatment Modality

Study	Combination therapy Events	Combination therapy Total	Monotherapy Events	Monotherapy Total	OR	95% CI	Weight
**PDE5i + Aspirin versus PDE5i**							
Bayraktar 2019	18	67	42	65	0.20	[0.10; 0.42]	7.8%
Heterogeneity: I^2 = NA%, p = NA	**18**	**67**	**42**	**65**	**0.20**	**[0.10; 0.42]**	**7.8%**
**PDE5i + Antioxidant versus PDE5i**							
Cavallini 2005	24	32	25	35	1.20	[0.41; 3.55]	6.6%
El-Wakeel 2019	26	30	20	29	2.92	[0.79; 10.89]	5.8%
Gallo 2020	67	100	53	99	1.76	[0.99; 3.13]	8.4%
Gentile 2004	2	20	0	20	5.54	[0.25; 123.08]	2.1%
Vicari 2010	5	53	32	53	0.07	[0.02; 0.20]	6.7%
Heterogeneity: I^2 = 88%, p < 0.01	**124**	**235**	**130**	**236**	**1.01**	**[0.24; 4.23]**	**29.6%**
**PDE5i + ACEi versus PDE5i**							
Chen 2012	5	31	2	31	2.79	[0.50; 15.62]	4.6%
Heterogeneity: I^2 = NA%, p = NA	**5**	**31**	**2**	**31**	**2.79**	**[0.50; 15.62]**	**4.6%**
**PDE5i + PDE5i versus PDE5i**							
Cui 2015	18	90	19	90	0.93	[0.45; 1.93]	7.9%
Heterogeneity: I^2 = NA%, p = NA	**18**	**90**	**19**	**90**	**0.93**	**[0.45; 1.93]**	**7.9%**
**PDE5i + Folic acid versus PDE5i**							
Hamidi Madani 2013	0	35	7	48	0.08	[0.00; 1.41]	2.4%
Heterogeneity: I^2 = NA%, p = NA	**0**	**35**	**7**	**48**	**0.08**	**[0.00; 1.41]**	**2.4%**
**PDE5i + A-blocker versus PDE5i**							
Jin 2011	41	137	19	66	1.06	[0.55; 2.02]	8.1%
Karami 2016	15	58	13	60	1.26	[0.54; 2.95]	7.5%
Kim 2017	33	134	24	152	1.74	[0.97; 3.13]	8.3%
Heterogeneity: I^2 = 0%, p = 0.52	**89**	**329**	**56**	**278**	**1.36**	**[0.93; 2.01]**	**23.9%**
**PDE5i + Pentoxifylline versus PDE5i**							
Kumar 2015	16	120	15	117	1.05	[0.49; 2.23]	7.8%
Law 2019	12	27	3	23	5.33	[1.27; 22.32]	5.4%
Heterogeneity: I^2 = 74%, p = 0.05	**28**	**147**	**18**	**140**	**2.10**	**[0.43; 10.23]**	**13.2%**
**PDE5i + Metformin versus PDE5i**							
Rey-Valzacchi 2012	9	14	1	6	9.00	[0.81; 100.14]	3.1%
Heterogeneity: I^2 = NA%, p = NA	**9**	**14**	**1**	**6**	**9.00**	**[0.81; 100.14]**	**3.1%**
**PDE5i + Testosterone versus PDE5i**							
Spitzer 2012	46	58	41	57	1.50	[0.63; 3.53]	7.4%
Heterogeneity: I^2 = NA%, p = NA	**46**	**58**	**41**	**57**	**1.50**	**[0.63; 3.53]**	**7.4%**
Heterogeneity: I^2 = 78%, p < 0.01	**337**	**1006**	**316**	**951**	**1.10**	**[0.66; 1.85]**	**100.0%**

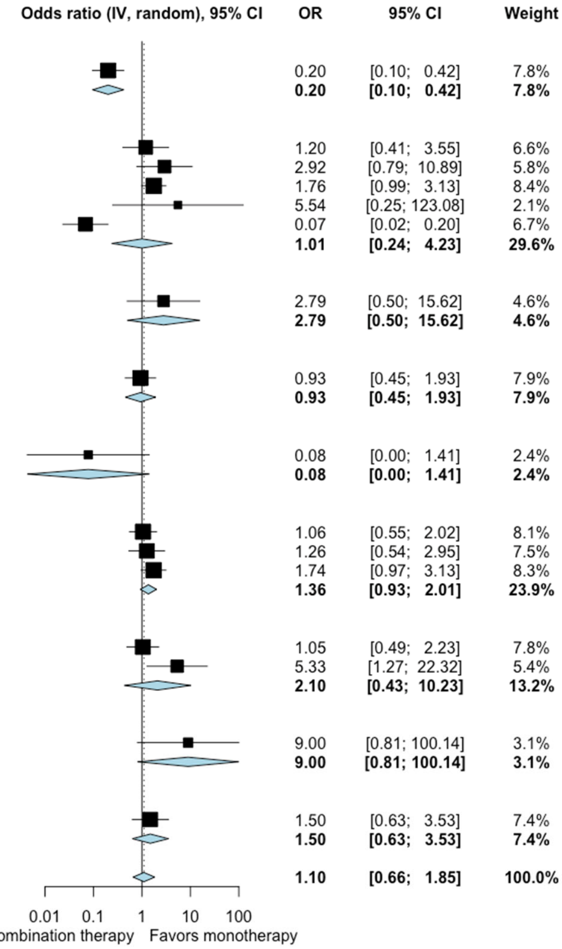

Number of adverse events with combination therapy versus monotherapy

Forest plot of the adverse events regarding different combination therapies versus PDE5i monotherapy. ACEi: angiotensin converting enzyme inhibitor; CI: confidence interval; ED: erectile dysfunction; IV: inverse variance; OR: odds ratio; PDE5i: phosphodiesterase type 5 inhibitors.

eAppendix 9. Efficacy Based on Impotent Subpopulation

Study	Combination therapy Patients	Combination therapy MD	Combination therapy SD	Monotherapy Patients	Monotherapy MD	Monotherapy SD	WMD	95% CI	Weight
**LUTS**									
Abolyosr 2013	50	4.6	3.38	50	4.6	3.32	-0.06	[-1.37; 1.25]	3.7%
Jin 2011	137	6.9	3.53	66	4.8	3.53	2.10	[1.06; 3.14]	4.1%
Jung 2008	49	10.4	4.02	52	8.1	4.02	2.30	[0.73; 3.87]	3.3%
Kaplan 2007	18	9.5	2.95	19	7.1	3.48	2.40	[0.33; 4.47]	2.6%
Karami 2016	58	7.6	2.10	60	7.8	1.38	-0.20	[-0.84; 0.44]	4.6%
Kim 2017	134	9.2	4.98	152	9.5	4.93	-0.32	[-1.47; 0.83]	3.9%
Liguori 2009	21	5.3	2.89	19	4.7	2.88	0.60	[-1.19; 2.39]	3.0%
Tuncel 2010	20	7.5	3.02	20	7.2	3.02	0.30	[-1.57; 2.17]	2.9%
Heterogeneity: I^2 = 72%, p < 0.01	**487**			**438**			**0.80**	**[-0.06; 1.65]**	**28.0%**
**Hypogonadism**									
Buvat 2011	72	3.6	7.40	66	3.1	7.30	0.50	[-1.95; 2.95]	2.2%
Shabsigh 2004	37	4.7	1.90	33	3.1	2.53	1.60	[0.54; 2.66]	4.0%
Shamloul 2005	10	2.2	1.09	10	0.5	0.80	1.70	[0.86; 2.54]	4.4%
Spitzer 2012	58	2.0	8.07	57	-0.2	8.07	2.20	[-0.75; 5.15]	1.8%
Heterogeneity: I^2 = 0%, p = 0.81	**177**			**166**			**1.61**	**[0.99; 2.23]**	**12.3%**
**Radical prostatectomy**									
Baccaglini 2020	36	-8.9	7.33	41	-12.4	7.33	3.50	[0.22; 6.78]	1.5%
Cavallini 2005	32	14.8	3.25	35	9.8	4.33	5.00	[3.18; 6.82]	2.9%
Engel 2011	13	17.7	2.87	7	9.3	4.23	8.40	[4.90; 11.90]	1.4%
Heterogeneity: I^2 = 53%, p = 0.12	**81**			**83**			**5.47**	**[3.11; 7.83]**	**5.8%**
**Monotherapy-resistant ED**									
Aversa 2003	10	7.4	2.88	10	1.0	2.88	6.40	[3.88; 8.92]	2.1%
Herrmann 2006	8	7.7	6.45	4	8.3	9.76	-0.60	[-11.16; 9.96]	0.2%
Rey-Valzacchi 2012	14	5.5	0.10	6	0.6	2.60	4.90	[2.82; 6.98]	2.6%
Shirai 2018	13	2.6	2.05	13	-0.0	3.21	2.65	[0.58; 4.72]	2.6%
Heterogeneity: I^2 = 52%, p = 0.10	**45**			**33**			**4.38**	**[2.37; 6.40]**	**7.6%**
**Other patients with ED**									
Bayraktar 2019	67	7.5	4.40	65	8.1	4.30	-0.60	[-2.08; 0.88]	3.4%
Chen 2012	31	8.7	1.45	31	6.6	1.37	2.07	[1.37; 2.77]	4.5%
Cui 2015	90	9.5	3.08	90	7.8	3.14	1.70	[0.79; 2.61]	4.3%
El Taieb 2019	27	9.8	2.75	27	7.7	2.75	2.10	[0.63; 3.57]	3.4%
El-Hamd 2020	30	13.7	1.86	30	12.3	1.53	1.40	[0.54; 2.26]	4.3%
El-Wakeel 2019	30	4.8	1.47	29	3.0	1.58	1.80	[1.02; 2.58]	4.4%
Gallo 2020	100	7.1	1.95	99	6.0	1.95	1.10	[0.56; 1.64]	4.7%
Gentile 2004	20	2.8	1.77	20	0.8	1.77	2.00	[0.90; 3.10]	4.0%
Hamidi Madani 2013	35	5.1	3.84	48	1.7	0.99	3.46	[2.16; 4.76]	3.7%
Kumar 2015	120	9.7	3.11	117	9.0	3.03	0.70	[-0.08; 1.48]	4.4%
Law 2019	27	1.8	4.63	23	2.5	4.63	-0.70	[-3.27; 1.87]	2.1%
Morano 2007	8	8.7	3.84	8	6.0	3.51	2.70	[-0.91; 6.31]	1.3%
Vicari 2010	53	5.4	8.07	53	3.9	8.01	1.50	[-1.56; 4.56]	1.7%
Heterogeneity: I^2 = 61%, p < 0.01	**638**			**640**			**1.52**	**[1.04; 2.00]**	**46.3%**
Heterogeneity: I^2 = 77%, p < 0.01	**1428**			**1360**			**1.76**	**[1.27; 2.24]**	**100.0%**
**Prediction interval**								**[-0.56; 4.08]**	

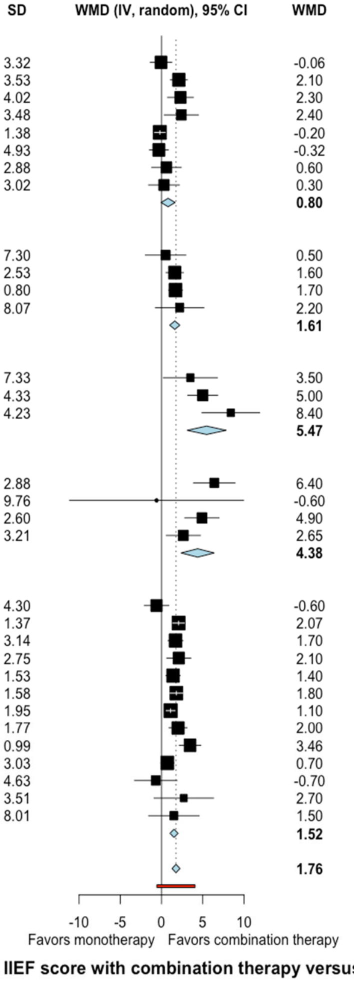

Forest plot of the mean difference in IIEF score with combination therapies versus PDE5i monotherapy in all identified subgroups with single study estimates. CI: confidence interval; ED: erectile dysfunction; IIEF: international index of erectile function; IV: inverse variance; LUTS: lower urinary tract symptoms; MD: mean difference; SD: standard deviation; WMD: weighted mean difference.

eAppendix 10. Adverse Events Based on Impotent Subpopulation

Study	Combination therapy Events	Combination therapy Total	Monotherapy Events	Monotherapy Total	Odds ratio (IV, random), 95% CI	OR	95% CI	Weight
**Other patients with ED**								
Bayraktar 2019	18	67	42	65		0.20	[0.10; 0.42]	7.8%
Chen 2012	5	31	2	31		2.79	[0.50; 15.62]	4.6%
Cui 2015	18	90	19	90		0.93	[0.45; 1.93]	7.9%
El-Wakeel 2019	26	30	20	29		2.92	[0.79; 10.89]	5.8%
Gallo 2020	67	100	53	99		1.76	[0.99; 3.13]	8.4%
Gentile 2004	2	20	0	20		5.54	[0.25; 123.08]	2.1%
Hamidi Madani 2013	0	35	7	48		0.08	[0.00; 1.41]	2.4%
Kumar 2015	16	120	15	117		1.05	[0.49; 2.23]	7.8%
Law 2019	12	27	3	23		5.33	[1.27; 22.32]	5.4%
Vicari 2010	5	53	32	53		0.07	[0.02; 0.20]	6.7%
Heterogeneity: I^2 = 85%, p < 0.01	**169**	**573**	**193**	**575**		**0.89**	**[0.38; 2.09]**	**58.9%**
**Radical prostatectomy**								
Cavallini 2005	24	32	25	35		1.20	[0.41; 3.55]	6.6%
Heterogeneity: I^2 = NA%, p = NA	**24**	**32**	**25**	**35**		**1.20**	**[0.41; 3.55]**	**6.6%**
**LUTS**								
Jin 2011	41	137	19	66		1.06	[0.55; 2.02]	8.1%
Karami 2016	15	58	13	60		1.26	[0.54; 2.95]	7.5%
Kim 2017	33	134	24	152		1.74	[0.97; 3.13]	8.3%
Heterogeneity: I^2 = 0%, p = 0.52	**89**	**329**	**56**	**278**		**1.36**	**[0.93; 2.01]**	**23.9%**
**Monotherapy-resistant ED**								
Rey-Valzacchi 2012	9	14	1	6		9.00	[0.81; 100.14]	3.1%
Heterogeneity: I^2 = NA%, p = NA	**9**	**14**	**1**	**6**		**9.00**	**[0.81; 100.14]**	**3.1%**
**Hypogonadism**								
Spitzer 2012	46	58	41	57		1.50	[0.63; 3.53]	7.4%
Heterogeneity: I^2 = NA%, p = NA	**46**	**58**	**41**	**57**		**1.50**	**[0.63; 3.53]**	**7.4%**
Heterogeneity: I^2 = 78%, p < 0.01	**337**	**1006**	**316**	**951**		**1.10**	**[0.66; 1.85]**	**100.0%**

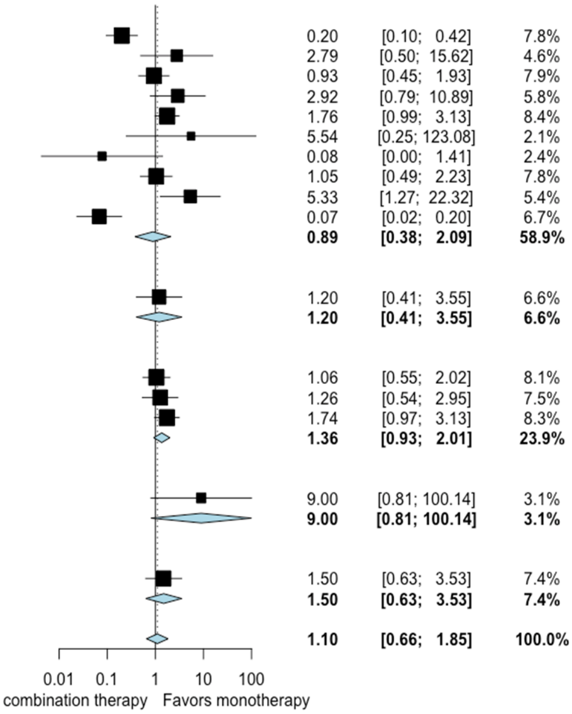

Number of adverse events with combination therapy versus monotherapy

Forest plot of the adverse events with combination therapies versus PDE5i monotherapy in all identified subgroups. CI: confidence interval; ED: erectile dysfunction; IV: inverse variance; LUTS: lower urinary tract symptoms; OR: odds ratio.

©2021 Mykoniatis I et al. *JAMA Network Open*

eAppendix 11. Subgroup Analysis According to PDE5i Response

Study	Combination therapy Patients	Combination therapy MD	Combination therapy SD	Monotherapy Patients	Monotherapy MD	Monotherapy SD	WMD	95% CI	Weight
**PDE5i responders**									
Abolyosr 2013	50	4.6	3.38	50	4.6	3.32	-0.06	[-1.37; 1.25]	3.7%
Baccaglini 2020	36	-8.9	7.33	41	-12.4	7.33	3.50	[0.22; 6.78]	1.5%
Bayraktar 2019	67	7.5	4.40	65	8.1	4.30	-0.60	[-2.08; 0.88]	3.4%
Cavallini 2005	32	14.8	3.25	35	9.8	4.33	5.00	[3.18; 6.82]	2.9%
Chen 2012	31	8.7	1.45	31	6.6	1.37	2.07	[1.37; 2.77]	4.5%
Cui 2015	90	9.5	3.08	90	7.8	3.14	1.70	[0.79; 2.61]	4.3%
El Taieb 2019	27	9.8	2.75	27	7.7	2.75	2.10	[0.63; 3.57]	3.4%
El-Hamd 2020	30	13.7	1.86	30	12.3	1.53	1.40	[0.54; 2.26]	4.3%
El-Wakeel 2019	30	4.8	1.47	29	3.0	1.58	1.80	[1.02; 2.58]	4.4%
Engel 2011	13	17.7	2.87	7	9.3	4.23	8.40	[4.90; 11.90]	1.4%
Gallo 2020	100	7.1	1.95	99	6.0	1.95	1.10	[0.56; 1.64]	4.7%
Gentile 2004	20	2.8	1.77	20	0.8	1.77	2.00	[0.90; 3.10]	4.0%
Hamidi Madani 2013	35	5.1	3.84	48	1.7	0.99	3.46	[2.16; 4.76]	3.7%
Jin 2011	137	6.9	3.53	66	4.8	3.53	2.10	[1.06; 3.14]	4.1%
Jung 2008	49	10.4	4.02	52	8.1	4.02	2.30	[0.73; 3.87]	3.3%
Kaplan 2007	18	9.5	2.95	19	7.1	3.48	2.40	[0.33; 4.47]	2.6%
Karami 2016	58	7.6	2.10	60	7.8	1.38	-0.20	[-0.84; 0.44]	4.6%
Kim 2017	134	9.2	4.98	152	9.5	4.93	-0.32	[-1.47; 0.83]	3.9%
Kumar 2015	120	9.7	3.11	117	9.0	3.03	0.70	[-0.08; 1.48]	4.4%
Law 2019	27	1.8	4.63	23	2.5	4.63	-0.70	[-3.27; 1.87]	2.1%
Liguori 2009	21	5.3	2.89	19	4.7	2.88	0.60	[-1.19; 2.39]	3.0%
Morano 2007	8	8.7	3.84	8	6.0	3.51	2.70	[-0.91; 6.31]	1.3%
Shamloul 2005	10	2.2	1.09	10	0.5	0.80	1.70	[0.86; 2.54]	4.4%
Spitzer 2012	58	2.0	8.07	57	-0.2	8.07	2.20	[-0.75; 5.15]	1.8%
Tuncel 2010	20	7.5	3.02	20	7.2	3.02	0.30	[-1.57; 2.17]	2.9%
Vicari 2010	53	5.4	8.07	53	3.9	8.01	1.50	[-1.56; 4.56]	1.7%
Heterogeneity: $I^2 = 76\%$, $p < 0.01$	**1274**			**1228**			**1.55**	**[1.06; 2.04]**	**86.2%**
**PDE5i non-responders**									
Buvat 2011	72	3.6	7.40	66	3.1	7.30	0.50	[-1.95; 2.95]	2.2%
Aversa 2003	10	7.4	2.88	10	1.0	2.88	6.40	[3.88; 8.92]	2.1%
Herrmann 2006	8	7.7	6.45	4	8.3	9.76	-0.60	[-11.16; 9.96]	0.2%
Rey-Valzacchi 2012	14	5.5	0.10	6	0.6	2.60	4.90	[2.82; 6.98]	2.6%
Shabsigh 2004	37	4.7	1.90	33	3.1	2.53	1.60	[0.54; 2.66]	4.0%
Shirai 2018	13	2.6	2.05	13	-0.0	3.21	2.65	[0.58; 4.72]	2.6%
Heterogeneity: $I^2 = 75\%$, $p < 0.01$	**154**			**132**			**3.02**	**[1.17; 4.87]**	**13.8%**
Heterogeneity: $I^2 = 77\%$, $p < 0.01$	**1428**			**1360**			**1.76**	**[1.27; 2.24]**	**100.0%**

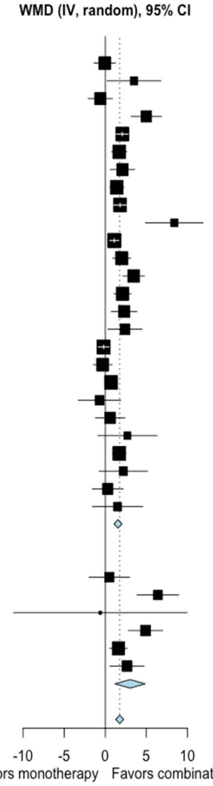

Mean difference in IIEF score with combination therapy versus monotherapy

Subgroup analysis comparing PDE5i responders and non-responders for the mean difference in IIEF score with combination therapy versus PDE5i monotherapy. CI: confidence interval; ED: erectile dysfunction; IIEF: international index of erectile function; IV: inverse variance; PDE5i: phosphodiesterase type 5 inhibitors; RCT: randomized controlled trial; WMD: weighted mean difference.

eAppendix 12. Sensitivity Analysis Including Placebo-Controlled RCTs

Study	Combination therapy Patients	Combination therapy MD	Combination therapy SD	Monotherapy Patients	Monotherapy MD	Monotherapy SD	WMD	95% CI	Weight
**PDE5i + Testosterone versus PDE5i**									
Buvat 2011	72	3.6	7.40	66	3.1	7.30	0.50	[-1.95; 2.95]	5.9%
Aversa 2003	10	7.4	2.88	10	1.0	2.88	6.40	[3.88; 8.92]	5.7%
Shabsigh 2004	37	4.7	1.90	33	3.1	2.53	1.60	[0.54; 2.66]	9.0%
Spitzer 2012	58	2.0	8.07	57	-0.2	8.07	2.20	[-0.75; 5.15]	4.9%
Heterogeneity: I^2 = 78%, p < 0.01	**177**			**166**			**2.60**	**[0.32; 4.87]**	**25.5%**
**PDE5i + Antioxidant versus PDE5i**									
Cavallini 2005	32	14.8	3.25	35	9.8	4.33	5.00	[3.18; 6.82]	7.3%
El Taieb 2019	27	9.8	2.75	27	7.7	2.75	2.10	[0.63; 3.57]	8.1%
El-Hamd 2020	30	13.7	1.86	30	12.3	1.53	1.40	[0.54; 2.26]	9.4%
Gentile 2004	20	2.8	1.77	20	0.8	1.77	2.00	[0.90; 3.10]	8.9%
Morano 2007	8	8.7	3.84	8	6.0	3.51	2.70	[-0.91; 6.31]	3.9%
Shirai 2018	13	2.6	2.05	13	-0.0	3.21	2.65	[0.58; 4.72]	6.7%
Heterogeneity: I^2 = 61%, p = 0.03	**130**			**133**			**2.45**	**[1.46; 3.45]**	**44.3%**
**PDE5i + Folic acid versus PDE5i**									
Hamidi Madani 2013	35	5.1	3.84	48	1.7	0.99	3.46	[2.16; 4.76]	8.5%
Heterogeneity: I^2 = NA%, p = NA	**35**			**48**			**3.46**	**[2.16; 4.76]**	**8.5%**
**PDE5i + Atorvastatin versus PDE5i**									
Herrmann 2006	8	7.7	6.45	4	8.3	9.76	-0.60	[-11.16; 9.96]	0.7%
Heterogeneity: I^2 = NA%, p = NA	**8**			**4**			**-0.60**	**[-11.16; 9.96]**	**0.7%**
**PDE5i + A-blocker versus PDE5i**									
Kim 2017	134	9.2	4.98	152	9.5	4.93	-0.32	[-1.47; 0.83]	8.8%
Heterogeneity: I^2 = NA%, p = NA	**134**			**152**			**-0.32**	**[-1.47; 0.83]**	**8.8%**
**PDE5i + Pentoxifylline versus PDE5i**									
Law 2019	27	1.8	4.63	23	2.5	4.63	-0.70	[-3.27; 1.87]	5.6%
Heterogeneity: I^2 = NA%, p = NA	**27**			**23**			**-0.70**	**[-3.27; 1.87]**	**5.6%**
**PDE5i + Metformin versus PDE5i**									
Rey-Valzacchi 2012	14	5.5	0.10	6	0.6	2.60	4.90	[2.82; 6.98]	6.7%
Heterogeneity: I^2 = NA%, p = NA	**14**			**6**			**4.90**	**[2.82; 6.98]**	**6.7%**
Heterogeneity: I^2 = 76%, p < 0.01	**525**			**532**			**2.32**	**[1.41; 3.23]**	**100.0%**

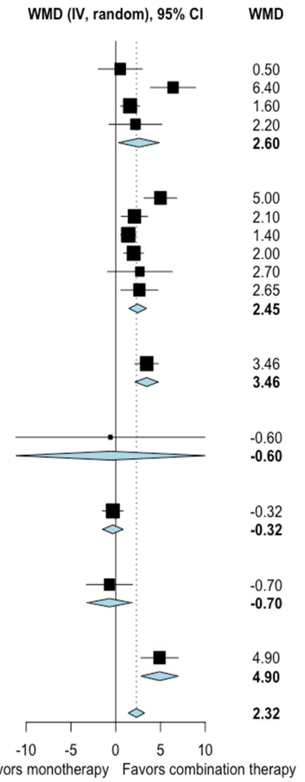

Mean difference in IIEF score with combination therapy versus monotherapy

Sensitivity analysis including placebo controlled RCTs for the mean difference in IIEF score with combination therapy versus PDE5i monotherapy. CI: confidence interval; ED: erectile dysfunction; IIEF: international index of erectile function; IV: inverse variance; MD: mean difference; PDE5i: phosphodiesterase type 5 inhibitors; RCT: randomized controlled trial; SD: standard deviation; WMD: weighted mean difference.

eAppendix 13. Sensitivity Analysis Including Studies at Low Risk of Bias

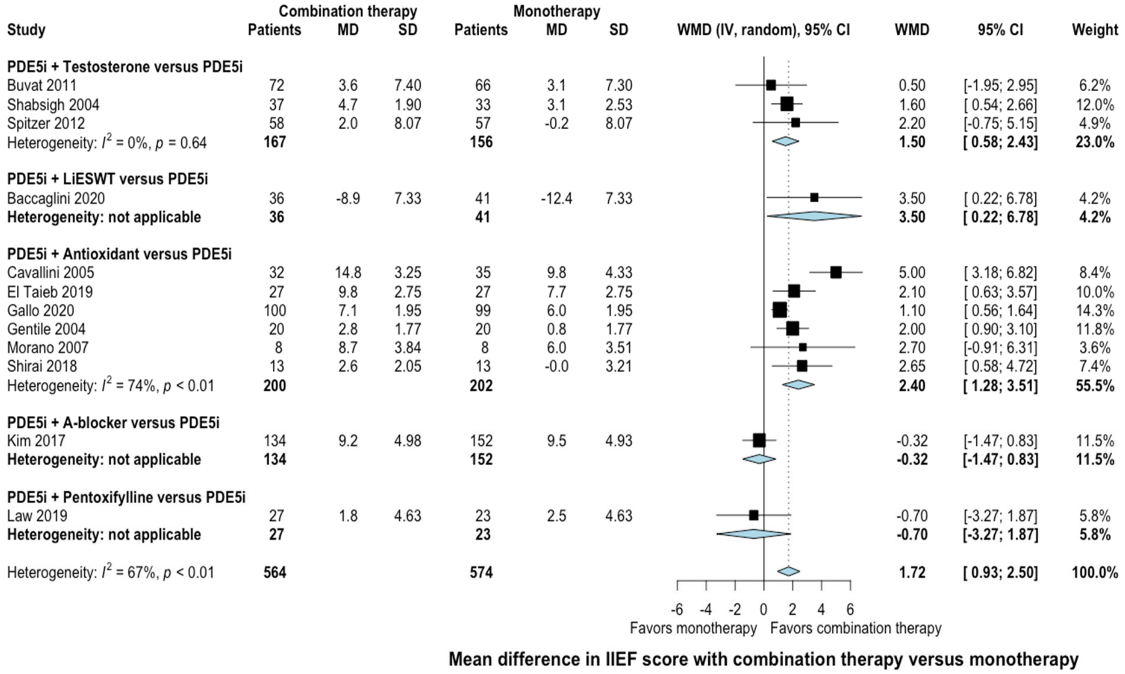

Sensitivity analysis including studies at low risk of bias for the mean difference in IIEF score with combination therapy versus PDE5i monotherapy. CI: confidence interval; ED: erectile dysfunction; IIEF: international index of erectile function; IV: inverse variance; MD: mean difference; PDE5i: phosphodiesterase type 5 inhibitors; RCT: randomized controlled trial; SD: standard deviation; WMD: weighted mean difference.

eAppendix 14. Grading of Evidence

Combination therapy versus monotherapy

Certainty assessment							№ of patients		Effect		Certainty	Importance
**№ of studies**	**Study design**	**Risk of bias**	**Inconsistency**	**Indirectness**	**Imprecision**	**Other considerations**	**Combination therapy**	**Monotherapy**	**Relative (95% CI)**	**Absolute (95% CI)**		
**Mean IIEF change from baseline**												
32	randomized trials	not serious	serious [a]	serious [b]	not serious	dose response gradient	1428	1360	Not applicable	MD **1.76 higher** (1.27 higher to 2.24 higher)	⊕⊕⊕◯ MODERATE	CRITICAL
**Number of adverse events**												
16	randomized trials	not serious	serious [a]	serious [b]	not serious	dose response gradient	337/1006 (33.5%)	316/951 (33.2%)	**OR 1.10** (0.66 to 1.85)	**21 more per 1,000** (from 85 fewer to 147 more)	⊕⊕⊕◯ MODERATE	CRITICAL

CI: Confidence interval; MD: Mean difference; OR: Odds ratio

Explanations

a. statistically significant heterogeneity among studies
b. different study populations among studies
